# Type I interferon response in astrocytes promotes brain metastasis by enhancing monocytic myeloid cell recruitment

**DOI:** 10.1038/s41467-023-38252-8

**Published:** 2023-05-06

**Authors:** Weili Ma, Maria Cecília Oliveira-Nunes, Ke Xu, Andrew Kossenkov, Benjamin C. Reiner, Richard C. Crist, James Hayden, Qing Chen

**Affiliations:** 1 Immunology, Microenvironment and Metastasis Program, The Wistar Institute, Philadelphia, PA 19104 USA; 2 MD/PhD Program, Boston University School of Medicine, Boston, MA 02215 USA; 3 Gene Expression & Regulation Program, The Wistar Institute, Philadelphia, PA 19104 USA; 4 Department of Psychiatry, Perelman School of Medicine, The University of Pennsylvania, Philadelphia, PA 19104 USA; 5 Imaging Shared Resource, The Wistar Institute, Philadelphia, PA 19104 USA; 6 Present Address: Carisma Therapeutics, Philadelphia, PA 19104 USA

**Keywords:** Metastasis, Cancer microenvironment, CNS cancer, Tumour immunology

## Abstract

Cancer metastasis to the brain is a significant clinical problem. Metastasis is the consequence of favorable interactions between invaded cancer cells and the microenvironment. Here, we demonstrate that cancer-activated astrocytes create a sustained low-level activated type I interferon (IFN) microenvironment in brain metastatic lesions. We further confirm that the IFN response in astrocytes facilitates brain metastasis. Mechanistically, IFN signaling in astrocytes activates C-C Motif Chemokine Ligand 2 (CCL2) production, which further increases the recruitment of monocytic myeloid cells. The correlation between CCL2 and monocytic myeloid cells is confirmed in clinical brain metastasis samples. Lastly, genetically or pharmacologically inhibiting C-C Motif Chemokine Receptor 2 (CCR2) reduces brain metastases. Our study clarifies a pro-metastatic effect of type I IFN in the brain even though IFN response has been considered to have anti-tumor effects. Moreover, this work expands our understandings on the interactions between cancer-activated astrocytes and immune cells in brain metastasis.

## Abstract (web summary)

Astrocytes can influence several steps of the metastatic process in the brain. Here the authors show that type I interferon response in astrocytes facilitates brain metastasis by increasing recruitment of tumor promoting monocytic myeloid cells.

## Introduction

Brain metastasis is the most ominous form of relapse in cancer patients, which is associated with poor prognosis and almost invariably lethal. The most common sources of brain metastasis are lung, breast carcinoma, and melanoma. Despite significant improvements in cancer treatment, current therapies have limited efficacy in brain metastases^1–5^. Consequently, brain metastasis becomes a significant clinical challenge in patients who have survived the primary tumor and extracranial metastases. Therefore, there is an urgent need to overcome this challenge by identifying mechanistic insights, prognostic markers, and novel therapeutic targets in brain metastasis studies.

Upon arrival in the distal organs, invaded cancer cells passively adapt to the new microenvironment. Meanwhile, the cancer cells actively modify the stromal cells to create a metastasis-specific microenvironment^6,7^. In the brain, astrocytes are the most abundant stromal cells. Metastatic cancer cells induce astrogliosis by activating the surrounding astrocytes, marked by increased glial fibrillary acidic protein (GFAP) expression and cellular processes^8,9^. Of note, the versatile astrocytes have diverse functions. On one hand, astrocytes release the killing factor in the microenvironment to induce cancer cell apoptosis^10^. On the other hand, astrocytes have been shown to facilitate cancer cell survival, growth, and migration at different stages of metastatic outgrowth^10–17^. Interactions between cancer cells and astrocytes in the brain microenvironment are dynamic, complex, and far more inextricably linked.

Immune cells are the most studied microenvironmental cells in cancer. Multiple myeloid subpopulations have been shown to facilitate carcinogenesis and metastasis. Myeloid-derived suppressor cells (MDSC), including monocytic MDSC (M-MDSC) and polymorphonuclear MDSC (PMN-MDSC), are currently defined as pathologically activated monocytes and neutrophils, respectively^18–20^. Tumor-associated macrophages are either differentiated from recruited monocytes and M-MDSC from circulation or modified from tissue residential macrophages^21–23^. Modified by the tumor microenvironment, these flexible myeloid cells elicit immunosuppressive functions to promote tumor growth^18–22^. Our knowledge on the immune cells in brain metastatic lesions is very limited. The specialized brain–blood barrier (BBB) provides a highly selective permeability barrier to the entrance of immune cells^24^. Thus, the brain used to be defined as an immune-privileged organ. The immunotherapies were expected to be ineffective in brain tumors due to the limited drug delivery and immune responses in the brain. Patients with active brain metastatic lesions were invariably excluded from immunotherapy clinical trials. However, this concept has been revised. The brain has an immune-specialized rather than immune-privileged environment under pathological conditions^25^. Retrospective studies indicate that multiple immunotherapies improved overall survival in brain metastasis patients^26–30^. Therefore, immune cells are an important component in the brain microenvironments that regulate metastatic outgrowth.

Our current study detects chronic low-level type I interferon (IFN) activation in the cancer cells and the reciprocal astrocytes throughout the brain metastatic process. IFN responses in astrocytes promote brain metastasis by enhancing the recruitment of monocytic myeloid cells in both breast cancer and melanoma models. We further delineate that IFN signaling activates the production of C-C motif chemokine ligand 2 (CCL2) in reactive astrocytes. Genetically or pharmacologically targeting C-C motif chemokine receptor 2 (CCR2), the paired receptor of CCL2 on monocytic myeloid cells, decreases brain metastasis.

## Results

### Sustained low-level activation of type I IFN in brain metastasis

**Fig. 1:**
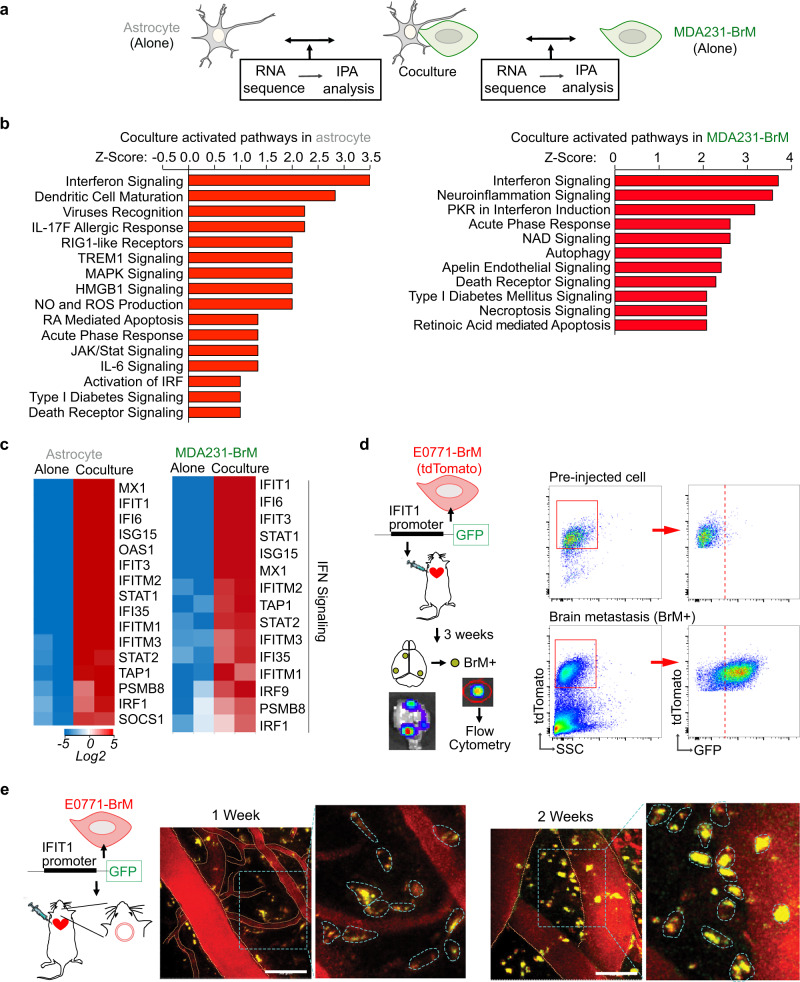
IFN signaling is activated in brain metastasis. **a**–**c** IFN signaling is activated in both human astrocytes and MDA231-BrM cells by coculture in vitro. *N* = 2 biologically independent experiments per condition. **a** Scheme of RNA sequencing experiment setup. **b** Ingenuity Pathway analysis (IPA) comparing BrM-induced changes in astrocytes and astrocyte-induced changes in BrM cells. **c** Heatmaps of genes in IFN signaling pathway from IPA in astrocytes and BrM cells. **d**, **e** IFN pathway is activated in brain metastatic lesions in vivo. IFIT1-GFP reporter structure is expressed in E0771-BrM cells. The reporter cells are injected into the experimental mice to track the activated IFN signaling in the brain metastasis microenvironment. **d** Macrometastatic lesions are isolated based on the luciferase signals in bioluminescent images (BLI), defined as BrM+ tissues. GFP expression in tdTomato^+^ BrM cells is detected by flow cytometry. **e** Representative images of intravital microscopy (IVM) observation of GFP expression in tdTomato^+^ BrM cells through an implanted cranial chamber. Vascular structure is outlined by orange dotted line. In the enlarged images, individual BrM cells are outlined by blue dotted line. Scale bar, 50 μm. Representative data shown of three biologically independent experiments.

Once extravasated into the brain parenchyma, the invaded cancer cells not only passively adapt to the new microenvironment but also actively modify the surrounding brain stromal cells. To better understand the mutual interactions between astrocytes and brain metastatic cancer cells, we set up in vitro coculture experiments and performed RNAseq in both cell types (Fig. 1a and Supplementary Fig. 1a, b). We used primary human astrocytes and MDA231-BrM cells, the brain metastatic derivatives (BrM) of human breast cancer MDA-MB-231 cells^31,32^, in the coculture experiments. BrM cells were stably labeled with fluorescent protein which allowed us to sort out cancer cells and astrocytes after coculture (Supplementary Fig. 1b, c). Compared to cultured alone condition, we unbiasedly screened the BrM cell-induced changes in astrocytes as well as the astrocyte-induced changes in BrM cells. Ingenuity Pathway Analysis (IPA) of significantly differentially expressed genes (false discovery rate (FDR) < 5%) allowed us to identify the signaling pathways activated by coculture. Of note, in both astrocytes and BrM cells, IFN signaling pathway was the top activated pathway (Fig. 1b, c).

We further validated whether IFN signaling was activated in the brain metastatic lesions in vivo. Since IFN signaling regulates immune response, we decided to use syngeneic brain metastasis mouse models for all the in vivo experiments. We performed in vivo selection to generate two mouse breast cancer BrM cells, E0771-BrM and A7C11-BrM, and melanoma Yumm1.7 BrM cells^17^. In brief, we injected the cancer cells into the left ventricle (intracardiac injection) of the experimental mice and the brain metastasis lesions were isolated to collect brain trophic metastatic cancer cells. After at least 2 rounds of in vivo selection, we were able to establish BrM cells to stably form brain metastases. All our mouse cancer cells were labeled with far-red luciferase and tdTomato for us to track the BrM cells (Supplementary Fig. 1c). In these tdTomato^+^ BrM cells, we stably expressed an IFN response reporter construct encoding the IFIT1 promoter fused with green fluorescent protein (IFIT1-GFP) (Fig. 1d). IFIT1 (Interferon Induced Protein with Tetratricopeptide Repeats 1) was one of the most upregulated IFN signaling genes in both BrM cells and astrocytes after coculture (Fig. 1c). 3 weeks after injecting the IFIT1-GFP reporter BrM cells into the experimental mice, we isolated the macrometastatic brain lesions and detected increased GFP expression in the tdTomato^+^ BrM cells compared to the preinjected cells (Fig. 1d and Supplementary Fig. 1d). Moreover, using intravital microscopy (IVM) observation through the implanted cranial chamber, we started to detect GFP signal in the IFIT1-GFP reporter BrM cells 1 week after cell inoculation (Fig. 1e). At this early time point, BrM cells were just extravasated into the brain parenchyma. The GFP expression sustained at the 2-week time point (Fig. 1e). Altogether, our data suggest that brain metastatic lesions maintain a sustained activated IFN microenvironment.

**Fig. 2:**
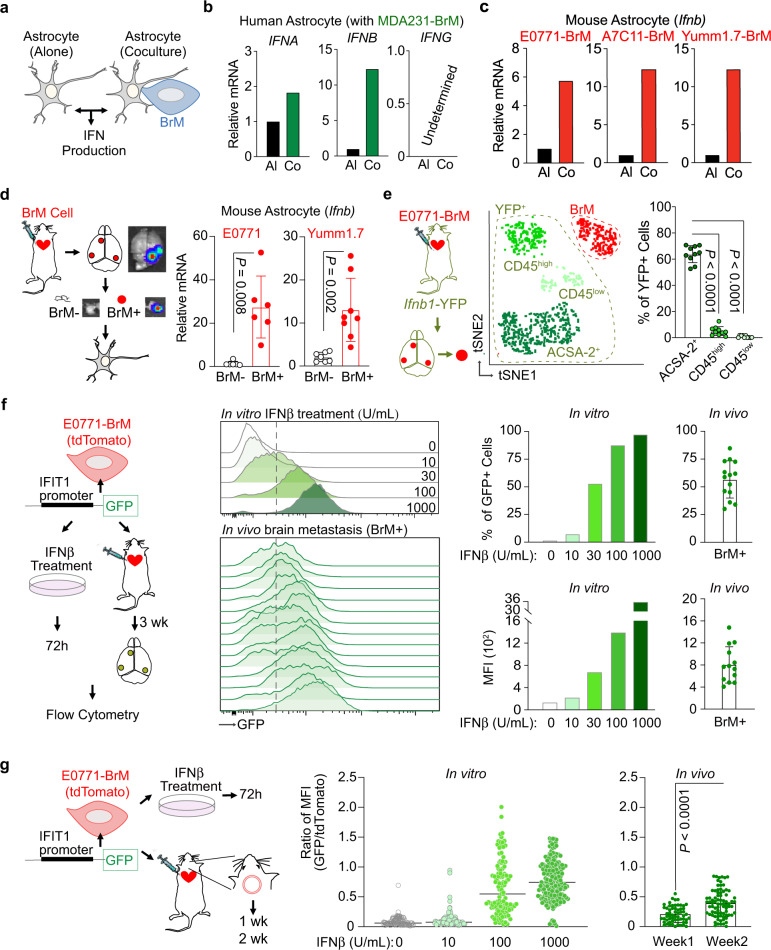
Low level of type I IFN signaling in brain metastasis microenvironment. **a**–**c** Astrocytes increase the production of IFNβ after coculture with BrM cells in vitro. **a** Scheme of experiment setup. **b** RT-PCR results of *IFN* genes in human astrocytes. **c** RT-PCR results of *Ifnb* genes in mouse astrocytes. Al cultured alone, Co cocultured. Representative data of three biologically independent experiments for each BrM cell. **d** Increased production of IFNβ in metastasis-activated astrocytes in vivo. From the experimental mice developed with brain metastasis, metastatic lesions (BrM+) and metastasis-free tissues (BrM−) are isolated based on the luciferase signals in BLI. Astrocytes are purified and *Ifnb* expression is detected by RT-PCR. Data are from merged samples from *n* = 3 biologically independent experiments and presented as mean ± S.D. E0771 model (BrM−: *n* = 4; BrM+: *n* = 6). Yumm1.7 model (BrM−: *n* = 7; BrM+: *n* = 8). Source data are provided as a Source data file. **e** Astrocytes are the major brain stromal cells producing IFNβ in brain metastasis in vivo. E0771-BrM cells are injected into *Ifnb1*-YFP reporter mice. Three subpopulations in YFP^+^ stromal cells are further defined: astrocytes (ACSA2^+^), infiltrated immune cells (CD45^high^), and brain resident microglia (CD45^low^). Representative tSNE includes tdTomato^+^ BrM cells (in red color) and 3 subpopulations in YFP^+^ stromal cells (in 3 shades of green) from a single BrM+ lesion. The percentage of each subpopulation in total YFP^+^ stromal cells is quantified in the bar graph. Data are merged 10 samples from 3 biologically independent experiments and presented as mean ± S.D. *P* values are from unpaired two-tailed *t* test. Source data are provided as a Source data file. **f**, **g** Low level of type I IFN activation in brain metastatic lesions in vivo. IFIT1-GFP reporter structure is expressed in E0771-BrM cells. The reporter cells treated with various concentrations of IFNβ in vitro and GFP expression is quantified (Representative data of 3 biologically independent experiments). From the brain metastatic lesions established by the reporter cells, the GFP expression in tdTomato^+^ BrM cells in vivo is quantified and compared to the IFNβ-treated cells. **f** Three weeks after injecting the reporter BrM cells, macrometastatic lesions are isolated. The percentage of GFP^+^ cells and mean fluorescent intensity (MFI) are quantified in tdTomato^+^ BrM cells. Data are merged 14 samples from 3 biologically independent experiments and presented as mean ± SD. **g** One week and 2 weeks after injection, the reporter BrM cells are observed by IVM through an implanted cranial chamber. The GFP signal is quantified and normalized by tdTomato^+^ signal from the same BrM cell. One-week data are merged 59 cells from 4 experimental mice. Two-week data are merged 80 cells from 3 experimental mice. Data are presented as mean ± S.D. *P* values are from unpaired two-tailed *t* test. Source data are provided as a Source data file.

We next evaluated the production of IFN cytokines in our in vitro cocultured cells (Fig. 2a). Human astrocytes expressed type I IFN (IFNα and IFNβ), but not type II IFN (IFNγ) (Fig. 2b). After coculture with MDA231-BrM cells, astrocytes increased the expression of type I IFNs, most significantly of IFNβ (Fig. 2b). We also set up coculture experiments using mouse astrocytes and 3 different mouse BrM cells: breast cancer E0771-BrM, A7C11-BrM and melanoma Yumm1.7-BrM cells. Consistently, mouse astrocytes did not express IFNγ (Supplementary Fig. 2a). IFNβ expression was significantly increased in mouse astrocytes cocultured with BrM cells (Fig. 2c) while no change was detected in IFNα expression after coculture (Supplementary Fig. 2a). For cancer cells, we did not detect the expression of IFNα, IFNβ or IFNγ in any of our human and mouse BrM cells (Supplementary Fig. 2b). Thus, in our in vivo brain metastasis models, we believe that the cellular sources of these IFN cytokines were from the brain stromal cells. Of note, these results support our previous work showing that gap junction communications between astrocytes and breast or lung cancer cells activate the production of type I IFN^33^. Here, we further setup indirect coculture experiment and confirmed that astrocyte–BrM contact was required for the enhanced IFNβ expression in astrocytes (Supplementary Fig. 2c). Overall, the results from these in vitro coculture experiments suggest that BrM-activated astrocytes enhance the expression of type I IFN, particularly IFNβ.

We further confirmed these observations in our in vivo brain metastasis mouse models. We used anti-astrocyte cell surface antigen-2 (ACSA-2) microbeads to purify astrocytes from the brain tissues of our experimental mice (Supplementary Fig. 2d)^34^. From the same brain, we isolated astrocytes from metastasis-free brain tissues (BrM- astrocytes) and brain metastatic lesions (BrM+ astrocytes) established by breast cancer E0771-BrM or melanoma Yumm1.7-BrM cells. Metastasis-free (BrM−) brain tissues were identified based on their low-luciferase signals (representative images in Fig. 2d). We also confirmed that these low-luciferase tissues did not have tdTomato^+^ BrM cells or CD45^high^ infiltrated immune cells by flow cytometry. Compared to BrM- astrocytes, BrM+ astrocytes had significantly higher expression of IFNβ (Fig. 2d). Consistent with our in vitro results, we did not detect any expression of IFNγ or change in IFNα production (Supplementary Fig. 2e). As an inflammatory cytokine, IFNβ is known to be produced by immune cells. Thus, we asked whether immune cells, in addition to astrocytes, produce IFNβ in brain metastasis. We used *Ifnb1*-YFP reporter mice and injected E0771-BrM. The basal YFP expression in the naive *Ifnb1*-YFP mice is not elevated compared to the matched wild type C57BL/6 mice (Supplementary Fig. 2f). In the brain metastatic lesions, a significantly higher proportion of brain stromal cells were YFP positive compared to BrM- brain tissues (Supplementary Fig. 2g). In these YFP^+^ stromal cells, about 60% were ACSA-2^+^ astrocytes (Fig. 2e). We also detected YFP signal in both CD45^low^ brain resident microglia and CD45^high^ infiltrated immune cells, but with much lower proportions (Fig. 2e). These data suggest that activated astrocytes are the major, but not the only, cellular source of IFNβ in the brain metastatic microenvironment. The much higher proportion of YFP^+^ astrocytes was consistent with the overall higher proportion of astrocytes in the brain metastasis microenvironment. Thus, we conclude that cancer-activated astrocytes contribute to create an activated IFN microenvironment by producing IFNβ in brain metastatic lesions.

Finally, we used a semi-quantitative approach to evaluate the activated type I IFN microenvironment in the brain metastatic lesions in vivo. We generated IFIT1-GFP reporter BrM cells to track and quantify IFN activation. We treated the reporter BrM cells with various doses of recombinant mouse IFNβ and measured the GFP signal by flow cytometry. We also established in vivo brain metastases using the reporter BrM cells. Using the in vitro treated cells as standard, we compared the GFP signals in the reporter BrM cells from brain metastatic lesions by the percentage of GFP^+^ cells and mean fluorescent intensity. Our results suggest that 3 weeks after BrM cell inoculation, the macrometastatic lesions have a low level of activated IFN microenvironment (10–100 Unit/mL of IFNβ) (Fig. 2f and Supplementary Fig. 2h). We further quantified the IFN activation at earlier stages of brain metastasis using cranial chamber/IVM observation. Using IFNβ-treated reporter cells as standard, we compared the GFP signal in the brain metastatic lesions 1 week and 2 weeks after BrM cell inoculation. We observed a consistent result showing that the GFP signals represented a low level of IFNβ activation in brain metastatic lesions (Fig. 2g and Supplementary Fig. 2i). Altogether, our data suggest that cancer-activated astrocytes produce IFNβ to create a chronic low level of activated type I IFN microenvironment throughout the brain metastasis process.

### Type I IFN response in astrocytes facilitates brain metastasis

**Fig. 3:**
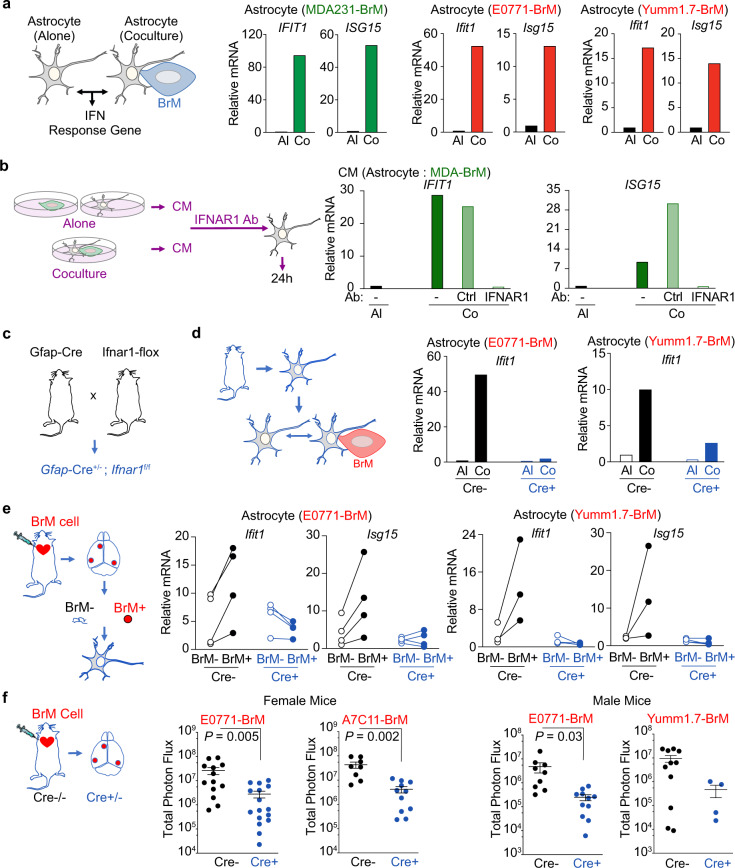
Type I IFN activation in astrocytes promotes brain metastasis. **a** Increased IFN response genes in astrocytes cocultured with BrM cells in vitro. Human astrocytes are cocultured with breast cancer MDA231-BrM cells. Mouse astrocytes are cocultured with breast cancer E0771-BrM and melanoma Yumm1.7-BrM. The expression levels of IFN response genes are measured by RT-PCR. Al cultured alone, Co cocultured. Representative data of 3 biologically independent experiments for each BrM cell. **b**–**e** Type I IFN signaling in astrocytes. **b** Human astrocytes are treated with conditioned media (CM) and type I IFN response genes are detected by RT-PCR. CM from astrocyte-BrM coculture (Co) or cultured alone (AL) are pretreated with either neutralizing antibody against IFNAR1 or the matched IgG control antibody (Ctrl). Representative data of 3 biologically independent experiments. **c** Astrocyte specific IFNAR1 knockout mice. **d** RT-PCR results of the type I IFN response gene, *Ifit1*, in the astrocytes cocultured with BrM cells in vitro. Primary cultured astrocytes are isolated from transgenic mice. Cre−, *Gfap*-Cre^−/−^; *Ifnar1*^f/f^ mice; Cre+, *Gfap*-Cre^+/−^; *Ifnar1*^f/f^ mice. Representative data of 3 biologically independent experiments for each BrM cell. **e** RT-PCR results of type I IFN response genes, *Ifit1* and *Isg15*, in the astrocytes from brain metastatic lesions in vivo. From the experimental mice developed with brain metastasis, the metastatic lesions (BrM+) and matched metastasis-free tissues (BrM−) are isolated based on the luciferase signals in BLI. Data are merged samples from 2 biologically independent experiments. E0771 model (4 pairs for both Cre+ and Cre−). Yumm1.7 model (Cre−: 3 pairs; Cre+: 4 pairs). Source data are provided as a Source data file. **f** Type I IFN signaling in astrocytes facilitates brain metastasis. Breast cancer E0771-BrM, A7C11-BrM and melanoma Yumm1.7-BrM are injected into Cre- or Cre+ mice. Quantification of brain lesions by BLI. Data are merged from 3 biologically independent experiments and presented as mean ± S.E.M. *P* values are from unpaired two-tailed *t* test. Female mice: E0771 model (Cre−: *n* = 13; Cre+: *n* = 16); A7C11 model (Cre−: *n* = 8; Cre+: *n* = 11). Male mice: E0771 model (Cre−: *n* = 9; Cre+: *n* = 16); Yumm1.7 model (Cre−: *n* = 11; Cre+: *n* = 4). Source data are provided as a Source data file.

Type I IFN responses in the brain play critical roles in physiological maintenance and multiple pathological processes, including viral infection and autoimmune diseases^35–37^. Our data indicate that brain metastasis has an activated type I IFN microenvironment. Thus, we focused on the effect of IFN activation in astrocytes during brain metastasis. In our in vitro coculture experiments, we first validated the IFN pathway genes identified in the astrocytes from our RNAseq results (Fig. 1c). Both breast cancer and melanoma BrM cells enhanced the expression of IFIT1 and Ubiquitin Like Modifier (ISG15) in the cocultured astrocytes (Fig. 3a and Supplementary Fig. 3a). We next asked whether the IFN activation in astrocytes was mediated by type I IFN, particularly IFNβ. We collected conditioned media (CM) from alone culture or cocultured MDA-BrM cells and human astrocytes. Treatment with cocultured CM activated IFN response genes (*IFIT1* and *ISG15*) in astrocytes (Supplementary Fig. 3b). The activated IFN response from cocultured CM was blocked by neutralizing antibodies of human IFNβ or type I IFN receptor, interferon α and β receptor subunit 1(IFNAR1) (Supplementary Fig. 3c and Fig. 3b). In addition, we established transgenic mice to knock out IFNAR1 specifically in astrocytes by crossbreeding GFAP-Cre and IFNAR1-flox mice (Fig. 3c). Astrocytes in *Gfap*-Cre^+/−^; *Ifnar*1^fl/fl^ (Gfap-Cre^+^) mice do not respond to type I IFN in the microenvironment while astrocytes from Gfap-Cre^−^ mice maintain normal response. We confirmed that depleting type I IFN response in astrocytes neither affected the growth of the primary cultured astrocytes, nor impacted the viability and number of astrocytes in the adult brain (Supplementary Fig. 3d, e). Using the primary cultured astrocytes from these transgenic mice, we confirmed that the BrM cell-induced IFIT1 and ISG15 expressions were inhibited in IFNAR1 knockout astrocytes in our in vitro co-culture experiments (Fig. 3d and Supplementary Fig. 3f).

Moreover, in our in vivo brain metastasis mouse models of both breast cancer and melanoma, we isolated astrocytes from brain metastatic lesions to detect the IFN activated genes. In Gfap-Cre^−^ mice, we detected a significant increase in IFIT1 and ISG15 expressions in BrM+ astrocytes, compared to BrM- astrocytes (Fig. 3e). These enhanced IFIT1 and ISG15 expressions were abrogated in Gfap-Cre^+^ mice (Fig. 3e). Thus, we confirmed the type I IFN activation in reactive astrocytes in brain metastatic lesions. Finally, in Gfap-Cre^+^ mice, we validated the effect of the astrocytic IFN response in brain metastatic outgrowth. In the indicated experiments, we used female mice for breast cancer BrM cells (E0771-BrM and A7C11-BrM) and male mice for melanoma Yumm1.7 BrM cells since these BrM cells were generated from female and male mice, respectively. In addition, we repeated the experiments using E0771-BrM cells in male mice. Our results showed that depleting IFNAR1 in astrocytes significantly decreased brain metastatic outgrowth of breast cancer and melanoma in both female and male mice (Fig. 3f). In these in vivo brain metastasis experiments, we used total photon flux of luciferase to quantify the brain metastatic burdens. We confirmed that the total photon flux was correlated with tumor regions quantified by immunohistochemical staining of tdTomato (Supplementary Fig. 3g). Of note, type I IFN has been reported to directly inhibit the growth of cancer cells^38^. However, our data indicate that brain metastasis has an activated type I IFN microenvironment and the astrocytic IFN response facilitates brain metastasis in vivo. To better understand this paradox, we investigated the effect of type I IFN on the growth of our mouse BrM cells. When BrM cell were treated with 10-100 units/mL INFβ, the equivalent range in brain metastatic lesions, we did not detect any change in cell growth. The inhibitory effect was observed only in the treatments with high concentrations of INFβ (Supplementary Fig. 3h). Altogether, our data suggest that the chronic low level of activated type I IFN microenvironment does not directly inhibit the growth of the metastatic cancer cells, but facilitates brain metastasis by activating IFN signaling in the surrounding astrocytes.

### Type I IFN activation in astrocytes increases myeloid cell infiltration

**Fig. 4:**
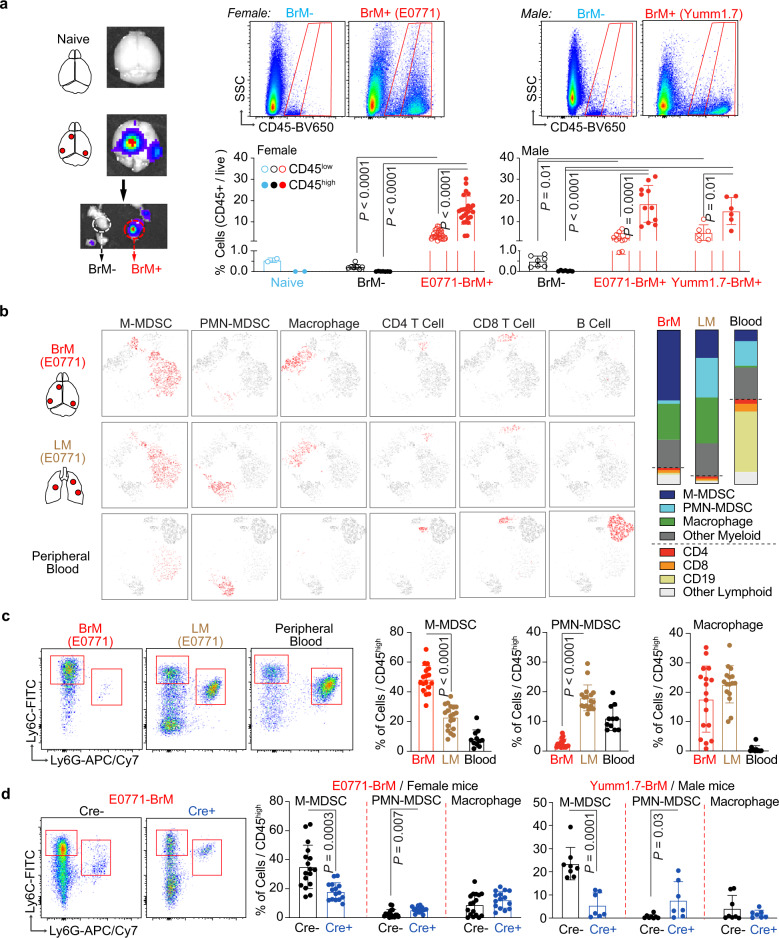
Type I IFN activation in astrocytes facilitate monocytic myeloid cell infiltration. **a** Immune cells in brain metastatic lesions in vivo. Immune cells, stained with CD45, are analyzed in brain tissues from the naive and experimental mice developed with brain metastasis. Metastatic lesions (BrM+) and metastasis-free tissues (BrM-) are isolated based on the luciferase signals in BLI. Percentages of CD45^low^ resident microglia and CD45^high^ infiltrated immune cells are quantified using flow cytometry. Data are merged samples from *n* = 3 biologically independent experiments presented as mean ± S.D (2 naive brain, 7 female BrM−, 24 female E0771 BrM+, 7 male BrM−, 11 male E0771 BrM+ and 6 male Yumm1.7 BrM+ samples). *P* values are from unpaired two-tailed *t* test. Source data are provided as a Source data file. **b**, **c** CD45^high^ infiltrated immune cells in brain metastatic lesions are further analyzed. **b** E0771-BrM cells are injected into experimental mice to establish brain or lung metastasis. Representative tSNE files of myeloid and lymphoid subpopulations of one brain metastasis lesion, merged lung metastatic samples from one experimental mouse and the blood sample from one brain metastasis-bearing mouse. M-MDSC, monocytic myeloid-derived suppressor cells; PMN-MDSC, polymorphonuclear MDSC. Bar graphs indicate the proportions of indicated immune subpopulations in CD45^high^ cells (total is 100%). **c** Dot plots show the representative flow profiles of MDSCs. Bar graphs show the percentages of myeloid subpopulations out of CD45^high^ cells. Data are merged samples from 4 biologically independent experiments (17 BrM, 19 LM and 11 blood samples). Data are presented as mean ± S.D. *P* values are from unpaired two-tailed *t* test. Source data are provided as a Source data file. **d** Type I IFN signaling in astrocytes facilitates monocytic myeloid cell infiltration. Breast cancer E0771-BrM and melanoma Yumm1.7-BrM cells are injected into transgenic mice with astrocyte specific IFNAR1 knock out. Cre−, *Gfap*-Cre^−/−^; *Ifnar1*^f/f^ mice; Cre+, *Gfap*-Cre^+/−^; *Ifnar1*^f/f^ mice. Dot plots show the representative flow profiles of MDSCs. Bar graphs show the percentages of myeloid subpopulations out of CD45^high^ cells. Data are merged samples from *n* = 3 biologically independent experiments and presented as mean ± S.D. *P* values are from unpaired two-tailed *t* test. E0771 model (Cre−: *n* = 17, Cre+: *n* = 15). Yumm1.7 model (Cre−: *n* = 8 -, Cre+: *n* = 7). Source data are provided as a Source data file.

We further investigated the underlying mechanism on how the astrocytic IFN activation could regulate brain metastasis. As inflammatory cytokines, IFNs are not only known to be produced by immune cells but also considered to be a central driver for immune response^39–42^. Here, we profiled the immune cells in the metastasis-free brain tissues (BrM−) and brain metastatic lesions (BrM+) in our mouse brain metastasis models. In consistence with previous reports, naive brain and BrM- tissues from brain metastasis-bearing mice only had CD45^low^ brain resident microglia with very limited CD45^high^ infiltrated immune cells from blood (Fig. 4a). In the BrM+ macrometastatic lesions established by breast cancer E0771-BrM and melanoma Yumm1.7-BrM cells, there were significantly more CD45^high^ infiltrated immune cells than CD45^low^ microglia (Fig. 4a). Further analyses showed that the majority of CD45^high^ cells were CD11b^+^ myeloid cells (Fig. 4b and Supplementary Fig. 4a). Finally, we used cell surface markers Ly6C, Ly6G and F4/80 to define 3 distinct myeloid populations: M-MDSC, PMN-MDSC and macrophages (Supplementary Fig. 4a)^18–20^. In addition to growing in the brain, E0771-BrM cells could form lung metastases when injected intravenously. The aggressive E0771-BrM cells allowed us to compare the immune cells in the brain and lung metastatic lesions established from the same cancer cells. We also collected blood samples from brain metastasis experimental mice to analyze circulating myeloid cells. Our results showed that M-MDSC was the predominant myeloid population in BrM+ lesions (Fig. 4b, c and Supplementary Fig. 4b–d). In contrast, both blood and lung metastasis samples contained more PMN-MDSC than M-MDSC (Fig. 4b, c and Supplementary Fig. 4b–d).

Our previous results showed that depleting type I IFN response in astrocytes reduced brain metastases (Fig. 3f). Thus, we tested the changes in the myeloid cell infiltration in the brain metastatic lesions in transgenic Gfap-Cre^+^ mice, using Gfap-Cre^−^ mice as the control. We detected a significant decrease in M-MDSC infiltration in the brain metastatic lesions from Gfap-Cre^+^ mice in which IFNAR1 was specifically knocked out in astrocytes (Fig. 4d). The proportion of PMN-MDSC remained very low and we did not detect significant changes in macrophage infiltration (Fig. 4d). MDSC in the tumor microenvironment have been shown to display potent immunosuppressive activity and promote tumor growth. Thus, we compared the expression of arginase-1 (Arg1), one of the signature immunosuppression genes, in the sorted M-MDSC from naive spleen, spleen from tumor-bearing mice as well as from brain metastatic lesions. Our data confirmed higher Arg1 expression in the splenic M-MDSC from tumor-bearing mice compared to naive mice. Moreover, we detected a dramatic increase of Arg1 expression in the M-MDSC from the brain metastatic lesions (Supplementary Fig. 4e). Altogether, our data indicate that type I IFN activation in astrocytes facilitates the infiltration of immunosuppressive M-MDSC into brain metastatic lesions.

### Type I IFN response activates CCL2 production in astrocytes to facilitate M-MDSC recruitment

**Fig. 5:**
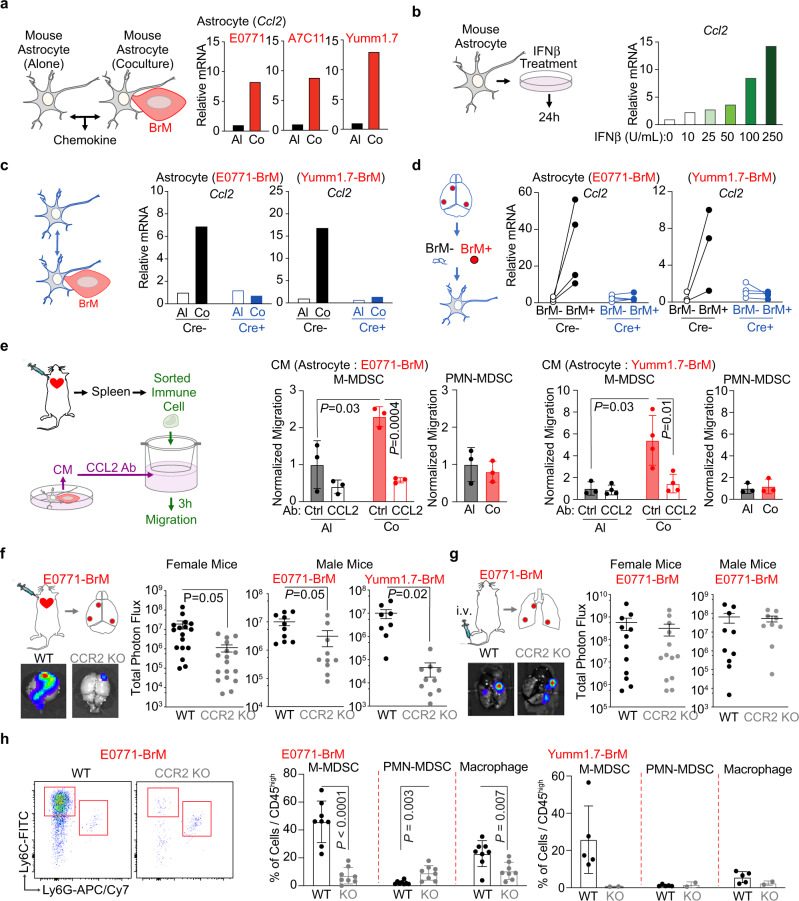
Type I IFN response activates CCL2 expression in astrocytes to facilitate myeloid cell infiltration and brain metastasis. **a** CCL2 expression is activated in astrocytes cocultured with BrM cells in vitro. Mouse astrocytes are cocultured with breast cancer E0771-BrM, A7C11-BrM and melanoma Yumm1.7-BrM. The expressions of CCL2 are measured by RT-PCR. Al, cultured alone astrocytes; Co, cocultured astrocytes. Representative data of 3 biologically independent experiments for each BrM cell. **b** IFNβ activates CCL2 expression in astrocytes in vitro. Mouse astrocytes cells are treated with various concentrations of IFNβ and the expressions of CCL2 are quantified by RT-PCR. *n* = 2 biologically independent experiments. **c**, **d** Type I IFN signaling activate CCL2 expression in astrocytes. **c** RT-PCR results of CCL2 expression in the astrocytes coculture with BrM cells in vitro. Primary cultured astrocytes are isolated from transgenic mice: Cre−, *Gfap*-Cre^−/−^; *Ifnfar1*^f/f^ mice; Cre+, *Gfap*-Cre^+/–^; *Ifnar1*^f/f^ mice. Representative data of 3 biologically independent experiments for each BrM cell. **d** RT-PCR results of CCL2 expression in the astrocytes from brain metastatic lesions in vivo. From the experimental mice developed brain metastasis, metastatic lesions (BrM+) and metastasis-free tissues (BrM−) are isolated based on the luciferase signals in BLI. Data are merged samples from 2 biologically independent experiments. E0771 model (4 pairs for both Cre+ and Cre−). Yumm1.7 model (Cre−: 3 pairs, Cre+: 4 pairs). Source data are provided as a Source data file. **e** CCL2 dependent migration of monocytic myeloid cells in vitro. In the top of migration chambers, M-MDSC and PMN-MDSC sorted from spleens of brain metastasis-bearing mice are loaded. Conditioned media (CM) from astrocyte-BrM coculture (Co) or cultured alone (AL) are pretreated with either neutralizing antibody against CCL2 (anti-CCL2) or the matched IgG control antibody (Ctrl) and loaded in the bottom of migration chambers. Data are the normalized migration of M-MDSC and PMN-MDSC. M-MDSC monocytic myeloid-derived suppressor cells, PMN-MDSC polymorphonuclear MDSC. Data are merged samples from 3 biologically independent experiments and presented as mean ± S.D. *P* values are from unpaired two-tailed *t* test. E0771 model (*n* = 3). Yumm1.7 model (*n* = 4). **f**, **h** Monocytic myeloid cell infiltration facilitates brain metastasis. **f** BrM cells are intracardially injected into CCR2 knockout (KO) and control wildtype (WT) mice. Quantification of brain lesions by BLI. BLI images show one representative sample in each group. Data are merged samples from *n* = 3 biologically independent experiments and presented as mean ± S.E.M. Female mice: E0771 model (WT and CCR2KO: *n* = 17). Male mice: E0771 model (WT and CCR2KO: *n* = 10). Yumm1.7 model (WT: *n* = 8, CCR2KO: *n* = 10). **g** BrM cells are intravenously injected into CCR2 KO and control WT female mice. Quantification of lung lesions by BLI. BLI images show one representative sample in each group. Data are merged samples from *n* = 3 biologically independent experiments and presented as mean ± S.E.M. Female mice (WT: *n* = 12; CCR2KO: *n* = 13). Male mice (WT: *n* = 10; CCR2KO: *n* = 9). **h** Percentages of myeloid subpopulations out of CD45^high^ cells in brain metastatic lesions. Data are merged samples from *n* = 3 biologically independent experiments and presented as mean ± S.D. E0771 model (WT and CCR2KO: *n* = 8). Yumm1.7 model (WT: *n* = 5, CCR2KO: *n* = 2). *P* values are from unpaired two-tailed *t* test. Source data are provided as a Source data file.

The recruitment of circulating immune cells is highly regulated by the chemokines in the tissues and the cognate chemokine receptors in the immune cells. Therefore, we hypothesized that the IFN activated-chemokine production in astrocytes attract M-MDSC into the brain metastatic lesions. We first tested the expression of several chemokines in the mouse astrocytes cocultured with BrM cells. CCL2-CCR2 pair, CXCL2 (C-X-C Motif Chemokine Ligand 2)-CXCR2 (C-X-C Motif Chemokine Receptor 2) pair and CXCL12-CXCR4 pair have been shown to regulate the recruitment of myeloid cells including monocyte/macrophage and MDSCs^19,20,43^. In our in vitro coculture experiments, CCL2 was the only commonly increased chemokine in astrocytes cocultured with E0771-BrM, A7C11-BrM and Yumm1.7-BrM cells (Fig. 5a and Supplementary Fig. 5a). We also treated mouse astrocytes with recombinant mouse IFNβ and showed that CCL2, but not CXCL2 or CXCL12, was upregulated in a dose dependent manner (Fig. 5b and Supplementary Fig. 5b). In these recombinant IFNβ experiments, we did not detect any change in the mRNA expression of *IFNβ* in treated astrocytes (Supplementary Fig. 5b). Moreover, one previous report shows that a pro-metastatic effect of signal transducer and activator of transcription 3 (STAT3) activation in a subpopulation of activated astrocytes^44^. In agreement with this work, we found that contact-independent cocultured astrocytes increased the production of interleukin 6 (IL-6) (Supplementary Fig. 2c), a cytokine which activates STAT3. Moreover, IL-6 signaling was another one of the enriched pathways in cocultured astrocytes (Fig. 1b). However, directly treating astrocytes with IFNβ activated STAT1, but not STAT3 (Supplementary Fig. 5c). Therefore, our data suggest that STAT3 is not directly activated by type I IFN response in astrocytes. Finally, in both in vitro and in vivo experimental models, depleting IFNAR1 in astrocytes abolished the BrM-induced CCL2 expression (Fig. 5c, d). Therefore, we conclude that type I IFN signaling enhances the expression of CCL2 in cancer-activated astrocytes.

Next, we used an in vitro migration assay to investigate whether M-MDSC recruitment depended on the enhanced CCL2 production from astrocytes. We tested the chemokine receptors on the surface of MDSCs isolated from spleens of our brain metastasis mouse models. Our flow cytometry data showed that M-MDSC expressed CCR2 and PMN-MDSC expressed CXCR2 (Supplementary Fig. 5d). Using a publicly available human clinical brain metastasis dataset and web bioinformatics tool^45^, we confirmed that in clinical brain metastasis samples, monocyte/macrophages had relatively higher expression of CCR2 and neutrophils expressed higher level of CXCR2 (Supplementary Fig. 5e). Next, we setup a functional migration assay to test the response of M-MDSC to the increased CCL2 from astrocytes. We used the MDSCs sorted from the spleens of the experimental mice with brain metastases. Conditioned media (CM) from BrM and astrocyte cocultured cells only increased the migration of M-MDSC but not PMN-MDSC, compared to the CM from cells cultured alone (Fig. 5e). Moreover, this enhanced migration from coculture CM was blocked by a neutralizing antibody against CCL2 (Fig. 5e). These data support that increased CCL2 production in IFN responsive astrocytes promotes M-MDSC migration.

We further performed in vivo brain metastasis experiments using CCR2 knockout mice to validate whether CCL2-dependent recruitment of M-MDSC contributed to brain metastasis. In CCR2 knockout mice, we detected a significant decrease in brain metastasis established by breast cancer E0771-BrM and melanoma Yumm1.7-BrM cells (Fig. 5f). In contrast, CCR2 depletion in the host mice did not affect lung metastasis established by E0771-BrM cells (Fig. 5g). This could be due to the high infiltration of PMN-MDSC in the lung metastatic lesions (Fig. 4c), which express CXCR2 chemokine receptor (Supplementary Fig. 5d). Moreover, we analyzed the infiltrated myeloid cells in the brain metastatic lesions and confirmed a decreased infiltration of M-MDSC in CCR2 knockout mice (Fig. 5h). In CCR2 knockout mice, we also detected a significantly decreased proportion of macrophages in the brain lesions (Fig. 5h). Of note, when the enhanced CCL2 was abolished in IFNAR1-depleted astrocytes, we only observed decreased infiltration of M-MDSC, but not macrophages in Gfap-Cre^+^ mice (Fig. 4d). These results hint at additional functions of CCR2 signaling in monocytes/macrophages besides migration^46^. Finally, we profiled dendritic cells (DC) in the brain metastatic lesions and investigated the potential effect of CCR2 knockout on DC infiltration. DC from the tumor microenvironment, in contrast to classic DC during inflammation, have been classified into various subpopulations based on their cell surface markers and functions^47^. In the brain metastatic lesions, we identified an average 22% CD11c^+^MHCII^high^ DC in infiltrated CD45^high^ immune cells and very low infiltration (~1%) of CD11c^+^MHCII^low^ CD11b^−^Ly6C^+^ plasmacytoid DC (pDC) (Supplementary Fig. 5f). We further broke down CD11c^+^MHCII^high^ DC into conventional type 1 DC (cDC1), conventional type 2 DC (cDC2), monocyte-derived DC (moDC)^47,48^. We detected significantly decreased moDC, but not other DC subpopulations in CCR2 KO mice (Supplementary Fig. 5g). These results are consistent with a previous report using CCR2KO mice in a lung carcinoma model^47^. Of note, although these moDC are able to uptake and present antigen, they share similar cell surface markers and immunosuppressive function as M-MDSC^47^. In the tumor microenvironment, M-MDSC have been reported to differentiate into macrophages and moDC^48^. Altogether, our data indicate that type I IFN activation in astrocytes promotes brain metastasis by increasing CCL2 production to recruit monocytic myeloid cells.

### CCL2-CCR2 as therapeutic target for brain metastasis

**Fig. 6:**
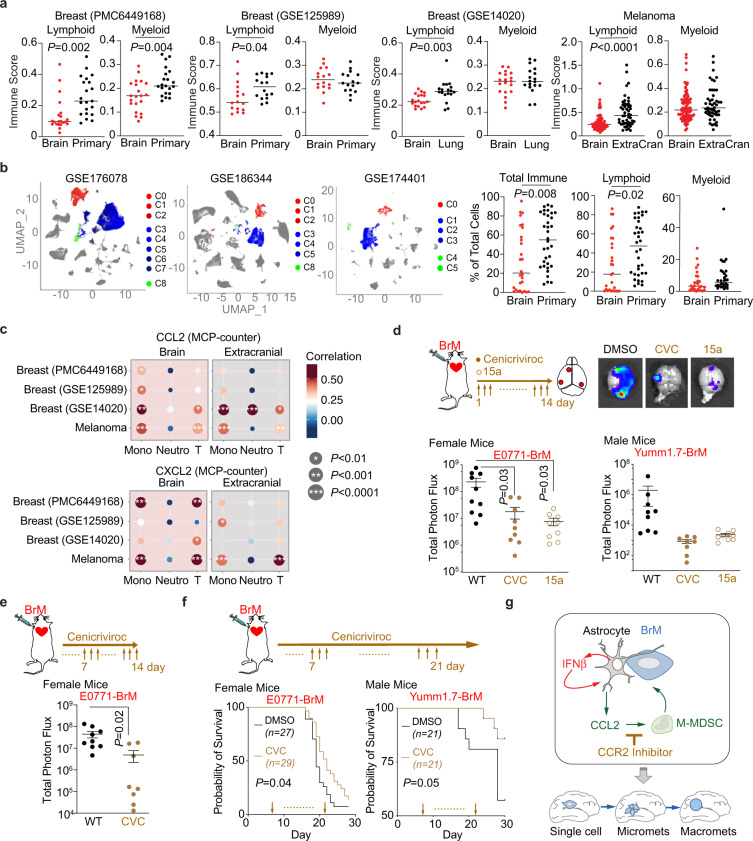
CCR2 is a therapeutic target to treat brain metastasis. **a** Bioinformatic analyses of immune cells in clinical samples. We include breast cancer PMC6449168 and GSE125989 (paired primary and brain metastatic datasets), GSE14020 (unpaired brain and lung metastatic samples), and melanoma melanoma EGAD00001005046 (paired and unpaired brain metastatic and extracranial tumors) in the analyses. Immune scores of lymphoid and myeloid immune subpopulations analyzed by CIBERSORT. *P* values are the results from unpaired two-tailed *t* test. PMC6449168: *n* = 22. GSE125989: *n* = 16. GSE14020: *n* = 19 Brain, *n* = 18 Lung. MD Melanoma: *n* = 88 Brain, *n* = 49 Extracranial. Source data are provided as a Source data file. **b** Single-cell RNAseq analysis of human clinical samples. After unsupervised clustering, we identified myeloid and lymphoid clusters from GSE174401 (melanoma BrM and extracranial), GSE176078 (primary breast cancer), and GSE186344 (Multiple BrM types) using gene expression signatures. Myeloid cells (red clusters), T/NK cells (blue clusters), B/Plasma cells (green clusters), other cancer/stromal cells (gray clusters). The proportions of total immune cells, myeloid and lymphoid cells were calculated out of total cells. *P* values are from unpaired two-tailed *t* test. Data points are individual samples from 3 datasets (28 brain, 34 extracranial). **c** Correlations of CCL2 or CXCL12 expression and immune scores of monocytic lineage (Mono), neutrophil (Neutro), and T lymphoid (T) subpopulations in brain metastatic and extracranial tumors analyzed by MCP-counter. Breast cancer PMC6449168 and GSE125989 (paired primary and brain metastatic datasets), GSE14020 (unpaired brain and lung metastatic samples), and melanoma EGAD00001005046 (paired and unpaired brain metastatic and extracranial tumors) are analyzed. *P* values are from unpaired two-tailed *t* test. **d–f** CCR2/CCR5 antagonist treatment decreases brain metastasis. Cenicriviroc (CVC) or 15a are systemically applied in the female experimental mice injected with breast cancer E0771-BrM cells and male mice injected with melanoma Yumm1.7-BrM cells. DMSO, vehicle control. **d** CVC or 15a are applied from day 1 after cancer cell inoculation. Brain metastases are quantified by bioluminescent imaging. BLI images show one representative sample in each group. Data are merged samples from 2 biologically independent experiments and presented as mean ± S.E.M (E0771 model: 10 DMSO, 9 CEN, 9 15a samples; Yumm1.7 model: 9 DMSO, 8 CEN, 9 15a samples). *P* values are from unpaired two-tailed *t* test. **e** CVC is applied from day 7 after cancer cell inoculation. Brain metastases are quantified by bioluminescent imaging. BLI images show one representative sample in each group. Data are merged samples from 2 biologically independent experiments and presented as mean ± S.E.M. *P* values are from unpaired two-tailed *t* test. **f** CVC is applied from day 7 after cancer cell inoculation. Probabilities of survival are tracked. Data are from merged samples of 2 biologically independent experiments. *P* values are from log-rank test. Source data are provided as a Source data file. **g** Schematic summary of the effect of type I IFN activation in astrocytes on brain metastasis. Cancer activated astrocytes, together with immune cells, create a sustained low level type I IFN activated microenvironment in the brain. IFN response in astrocytes promotes brain metastasis by secreting CCL2 to recruit monocytic myeloid cells. CCR2 is a therapeutic target to decrease brain metastasis by blocking myeloid cell infiltration.

We next explored whether we could target CCL2-dependent immune infiltration to treat brain metastasis. In publicly available human bulk RNAseq datasets, we first used multiple bioinformatics tools to analyze the immune populations in clinical samples from breast cancer and melanoma patients. For breast cancer, we included 2 cohorts with paired primary and brain metastatic tumor samples (PMC6449168 and GSE125989)^49,50^, and another dataset with unpaired brain and lung metastatic samples (GSE14020)^51,52^. For melanoma, we analyzed a recently published dataset containing paired and unpaired brain metastatic and extracranial tumors (EGAD00001005046)^53^. The immune score and composition were analyzed by recently developed bioinformatics tools, including CIBERSORT, MCP-counter and xCell^54–56^. Overall, brain metastatic samples had significantly lower immune scores, meaning significantly lower infiltration of immune cells, compared to primary or extracranial metastatic tumors (Supplementary Fig. 6a). In particular, the lower immune score in brain metastasis samples was mostly due to less lymphoid cell infiltration (Fig. 6a and Supplementary Fig. 6b). Moreover, we analyzed immune infiltrates in the publicly available human single cell RNAseq datasets of human brain metastases and extracranial tumors: a dataset containing brain metastases from various primary tumor origins (GSE186344)^57^; a cutaneous melanoma dataset containing both primary and brain metastases samples (GSE174401)^58^ and a comprehensive primary breast cancer dataset (GSE176078)^59^. In every individual dataset, we performed principal component analysis and unsupervised clustering (Fig. 6b). Cell type clusters were manually annotated using published gene signatures (Fig. 6b and Supplementary Fig. 6c). We focused on the immune cell clusters and calculated the proportions of total immune cells, lymphoid cells and myeloid cells in each sample. Consistently, we found a significant reduction of total immune infiltration in brain metastasis compared to extracranial samples (Fig. 6b). The reduced immune infiltration was mainly due to a significant decrease in the lymphoid population in brain metastasis (Fig. 6b). Thus, these data suggests that the brain tumor microenvironment more stringently restricts the accumulation of lymphoid cells compared to myeloid cells.

We further tested the correlation between CCL2 or CXCL12 expression and immune scores of various immune subpopulations from the bulk RNAseq datasets. Using both MCP-counter and CIBERSORT analyses, we got consistent results from all 4 clinical datasets showing that CCL2 expression was significantly correlated to the scores of monocytic lineage cells in brain metastasis samples (Fig. 6c and Supplementary Fig. 6d). This CCL2-monocytic lineage correlation was more robust in brain metastatic tumors than extracranial tumors. In contrast, CCL2 expression was not consistently correlated to immune scores of neutrophils or T lymphocytes among the 4 datasets (Fig. 6c and Supplementary Fig. 6d). When similar analyses were performed between CXCL2 expression and immune scores, we did not detect any correlation (Fig. 6c and Supplementary Fig. 6d). These analyses from clinical samples suggest that CCL2 is correlated with increased recruitment of monocytic immune cells into human brain metastatic lesions.

We next used our brain metastasis mouse model to test the therapeutic potential of blocking CCR2-dependent myeloid recruitment on brain metastatic outgrowth. We chose to use Cenicriviroc (CVC), a dual CCR2/CCR5 inhibitor being tested in clinical trials for a number of applications including liver fibrosis, HIV and COVID-19 (from https://clinicaltrials.gov: NCT01338883, NCT04334915, NCT03028740, NCT04500418)^60–62^. Another well-characterized and more specific CCR2 inhibitor 15a^63,64^ was also chosen to address the question. Daily administration of CVC or 15a from day 1 after cancer cell inoculation significantly decreased brain metastasis established from breast cancer E0771-BrM and melanoma Yumm1.7-BrM cells in both female and male experimental mice (Fig. 6d). Moreover, we confirmed that the infiltration of myeloid cells in brain metastatic lesions, including M-MDSC and macrophages, was significantly decreased after CVC treatment (supplementary Fig. 6e). These results are consistent with our brain metastasis assays using CCR2 knock out mice (Fig. 5f, h). Finally, to test the effect of CCR2 inhibitor on the extravasated cancer cells and their potential therapeutic benefit, we started the daily CVC treatment at day 7 after cancer cell inoculation. Even with this delayed treatment, we observed a similar decrease in brain metastatic burden (Fig. 6e) and a significant survival benefit in our very aggressive E0771-BrM breast cancer model and the Yumm1.7 melanoma model (Fig. 6f). These proof-of-concept experiments indicate that pharmacological inhibition of CCR2 may be a viable therapeutic strategy to control brain metastases.

## Discussion

As illustrated in the “seed and soil hypothesis”^6,7^, metastatic outgrowth in distal organs requires the complex interplay between cancer cells and the microenvironment. Here, we identify that the cancer activated astrocytes create an activated type I IFN microenvironment to facilitate brain metastasis (Fig. 6g).

Astrocytes are the most abundant and reactive brain stromal cells in brain metastatic lesions. Our previous work shows that gap junction communications between cocultured cancer cells and astrocytes mediate the cGAMP transfer from cancer cells to astrocytes, activating the cGAS-STING pathway to increase type I IFN production in astrocytes^11^. Here, we confirm that the reactive astrocytes, from the brain metastatic lesions in vivo, activated IFNβ production. Type I IFNs are inflammatory cytokines known to be produced by immune cells. As expected, we confirm that immune cells, including the recruited immune cells and residential microglia, also produce IFNβ. However, there are significantly higher numbers of IFNβ-producing astrocytes than immune cells in brain metastatic lesions. Of note, in our brain metastasis models, including 2 breast cancer and 1 melanoma, the cancer cells do not express any Type I or Type II IFN cytokines. Thus, the activated type I IFN microenvironment is created by the stromal cells in brain metastatic lesions. Altogether, our data demonstrate that in the brain metastatic lesions, reactive astrocytes are the major cellular source of IFNβ to create the activated type I IFN microenvironment.

Type I IFN response is considered to be a central driver for inflammation. In cancer, type I IFN has been considered to be beneficial by directly inhibiting cancer cell growth and indirectly promoting the anti-tumor effect of T cells^38–42^. In fact, type I IFN was the first immunotherapeutic agent approved by the FDA to treat cancer patients. However, with disappointing results, more and more studies suggest the dichotomous role of type I IFN in cancer and chronic inflammation^41^. In the brain, besides the expected function of controlling viral spread, type I IFN response has been reported to reduce neuroinflammation in experimental autoimmune encephalomyelitis and maintain synaptic plasticity^65–67^. In our current study on brain metastasis, we start to detect a low-level type I IFN signaling at the very early stage of the brain metastatic process after metastatic cancer cells extravasate into the brain parenchyma. This chronic low-level IFN signaling does not inhibit cancer cell growth but instead promotes brain metastasis by recruiting monocytic immune cells. Altogether, our study expands the knowledge on type I IFN production in the brain microenvironment and the IFN effect on brain metastasis. The current study also raises several questions to address in future studies including the role of type I IFN signaling in primary brain glioma and the effect of acute IFN signaling in brain metastasis. Of note, this identified mechanism is shared by breast cancer and melanoma, suggesting that the brain microenvironment elicits similar responses to the invaded cancer cells regardless of primary origins.

Previous studies show that reactive astrocytes secrete CCL2 in brain autoimmune diseases and, more relevantly to our current study, in primary brain glioma and breast cancer brain metastasis^68–70^. Here, we demonstrate that brain metastasis-activated astrocytes respond to the activated type I IFN microenvironment to increase CCL2 production. Of note, CCL2 can be produced by many other resident brain stromal cells including neurons, oligodendrocytes, endothelial cells, and microglia^71–75^. In the brain metastatic lesions, infiltrated immune cells, as well as cancer cells, can be additional cellular sources of CCL2. In fact, a previous report has shown that breast cancer cells increase CCL2 production in response to the exosomes secreted from the surrounding astrocytes^76^. The major cellular source of CCL2 needs to be further delineated. However, our data suggest that type I IFN activated CCL2 expression from the reactive astrocytes facilitate the infiltration of M-MDSC into the brain metastatic lesions.

The specialized BBB blocks the entrance of immune cells into the brain under physiological conditions. In brain metastatic lesions, the blood tumor barrier is more permissive^25^. However, our analyses of clinical samples show that in both breast cancer and melanoma, immune scores in brain metastases are still significantly lower compared to primary and extracranial metastatic tumors. This is due to the decreased lymphoid immune cell infiltration while myeloid cells exhibit a similar ability to get recruited into the brain metastatic lesions. These observations are consistent with previous brain metastasis studies on melanoma and lung carcinoma^53,77^. Infiltrated myeloid cells, such as tumor associated macrophages and neutrophils have been shown to suppress immune responses and promote brain metastasis^78–80^. Here, our data suggest that type I IFN response in astrocytes facilitates M-MDSC infiltration into the brain metastatic lesions. MDSC in the tumor microenvironment have been shown to promote tumor growth through multiple mechanisms. These cells display immunosuppressive function to the surrounding lymphocytes, NK cells, macrophages and dendritic cells; as well as promoting tumor angiogenesis by secreting soluble factors into the microenvironment (e.g., MMPs and VEGF) and differentiating into endothelial-like cells^75,81^. In our mouse brain metastasis models, M-MDSC but not PMN-MDSC are the major myeloid cells. Blocking the recruitment of M-MDSC inhibits brain metastasis. The high expression of arginase-1 suggests the potent immunosuppressive function of these cells in the brain metastatic lesions. However, more studies need to be done to understand, in the brain microenvironment, how M-MDSC promote brain metastatic outgrowth. Of note, our brain metastasis models are not spontaneous metastasis models. In the brain metastasis lesions, we detected <2% of CD8 T cells in the brain metastatic lesions. This data is in consistent with previous published work indicating extracranial tumors are required for CD8 T cell recruitment into the brain^82^. Thus, modified models need to be developed to fully understand the immunosuppressive functions of M-MDSC in brain metastasis. In addition, microglia, the brain resident myeloid cells, have been shown to elicit immune suppressive functions which facilitate brain metastasis^80,83,84^. Moreover, reviving phagocytosis in microglia can be used to treat brain metastasis^83,85^. Here, in both our breast cancer and melanoma brain metastasis mouse models, we detected significantly higher numbers of infiltrated immune cells (CD45^high^) compared to the resident brain microglia (CD45^low^). Thus, we focused on whether CCR2-dependent recruitment of M-MDSC facilitated brain metastasis. A related study on breast cancer metastasis shows that CX3CR1-mediated resident microglia migration promotes brain metastasis in the mouse models where resident microglia are the major immune population^83^. Collectively, both infiltrated M-MDSC and resident microglia appear to have pro-tumor functions in brain metastases. Further studies need to be done to compare/contrast these two populations and investigate the potential synergistic effect of CX3CR1 and CCR2 blockage.

In sum, our current study put invaded cancer cells, reciprocal reactive astrocytes, as well as recruited immune cells together to form a multi-dimensional system. These results will provide a more ‘complete’ picture of how cancer, the brain stroma and immune cells shape each other which eventually leads to metastatic outgrowth. By extension, breaking these interactions will provide additional directions to target and treat brain metastasis.

## Methods

### Mouse

All animal experiments were performed in accordance with protocols approved by the Wistar Institutional Animal Care and Use Committee. Animals were euthanized at appropriate experimental or humane endpoints. Humane endpoints include loss of motor function, moribund state, or loss of 20% total body weight. Animals were housed in temperature and humidity controlled environments on a 12-h light–dark cycle. C57BL/6J mice, B6.129-*Ifnb1*^*tm1Lky*^/J mice (Ifnb-YFP reporter), B6.129S4-Ccr2^tm1Ifc^/J (CCR2 knockout), B6.Cg-Tg(Gfap-Cre)77.6Mvs/2J, and B6(Cg)-Ifnar1^tm1.1Ees^/J mice were obtained from The Jackson Laboratory. GFAP-Cre line 77.6 mice are particularly useful for selective targeting of astrocytes since they are reported to have no Cre recombinase activity in postnatal or adult neural stem cells (or their progeny) from the hippocampus or other brain regions^86^. Tg(Gfap-Cre)77.6Mvs/2J and B6(Cg)-Ifnar1^tm1.1Ees^/J mice were crossed to generate *Gfap*-Cre^+/−^; *Ifnar1*^f/f^ and the control *Gfap*-Cre^−/−^; *Ifnar1*^f/f^ mice. The genotyping primers are listed in the Supplementary Table 4. Genotyping was performed by Transnetyx, Inc. All experimental animals were used at 5–6 weeks of age. Sex of the experimental mice are indicated in the individual experiments.

### Mouse experiment

BrM cells were generated by in vivo selection in mice, as previously described^87^. E0771 cells were ordered from CH3 Biosystems. Yumm1.7 cells were kindly provided by Dr. Ashani Weeraratna’s lab. A7C11 cells were kindly provided by Dr. Jose Conejo-Garcia’s lab. Cells were stably labeled with luciferase and tdTomato. 5 × 10^4^ cancer cells suspended in 100 μL PBS were injected into the left cardiac ventricle. Metastatic growth was monitored by bioluminescence imaging (BLI) using an IVIS SpectrumCT (PerkinElmer) after retro-orbital injection with D-luciferin (150 mg/kg) into the experimental mice. At the experimental endpoint, tumor regions were identified by ex vivo BLI. Isolated brain lesions were cultured as single-cell suspensions for 2 weeks, and fluorescent-labeled cancer cells were sorted on a BD FACSAria or MoFlo Astrios EQ.

For in vivo brain metastasis experiments, we followed previously described procedures^11^. In brief, 2.5 × 10^4^ E0771-BrM cells, 5 × 10^4^ A7C11-BrM or Yumm1.7-BrM cells suspended in 100 μL PBS were injected into the left cardiac ventricle. Fourteen to 21 days after injection, brain colonization was quantified by ex vivo BLI after retro-orbital injection with D-luciferin (150 mg/kg) into the experimental mice. The bioluminescent signal from whole brains was analyzed using Living Image Software (PerkinElmer). For isolating BrM+ tissues, the brain was harvested, cut into small tissues, and imaged by ex vivo BLI after retro-orbital injection with D-luciferin (150 mg/kg) into the experimental mice. In the indicated experiments, once the BrM+ tissue was identified, the matched BrM− tissue was picked from the other side of the symmetrical hemisphere. E0771-BrM cells were injected in both male and female mice, A7C11 BrM cells were injected in female mice and Yumm1.7-BrM cells were injected in male mice. For CCR2 antagonist treatment, Cenicriviroc was purchased from Medinoah and administered daily by intraperitoneal injections (30 mg/kg) for 14 days. Stock solutions were prepared in DMSO and diluted to working concentrations in corn oil (Spectrum Chemical MFG Corp) immediately prior to injections (final DMSO concentration <5%). DMSO was used as vehicle control. To generate lung metastases (LM), 5 × 10^5^ E0771-BrM cells suspended in 100 μL PBS were injected into the lateral tail vein. Mice were shaved and imaged twice a week. Successful LM indicated by high BLI signal in the chest was typically observed after 14 days. In the indicated experiments, mice were euthanized between day 18 and 20 for ex vivo BLI analysis. For positive control for IFN induction, *Ifnb*-YFP reporter mice received a single intraperitoneal injection of lipopolysaccharide (LPS, 5 mg/kg) for 24 h.

### Cell culture

Human MDA231-BrM and murine Yumm1.7-BrM cells were cultured in DMEM supplemented with 10% fetal bovine serum (FBS, Sigma) and 2 mM L-Glutamine. Murine A7C11-BrM and E0771-BrM cells were cultured in RPMI-1640 supplemented with 10% FBS and 2 mM L-glutamine. Human and mouse primary astrocytes were purchased from Sciencell (#1800 and #M1800-57, respectively) and cultured in astrocyte media specified by the supplier. Primary astrocytes were isolated from *Gfap*-Cre^+/−^; *Ifnar*1^fl/fl^ or *Gfap*-Cre^−/−^; *Ifnar1*^fl/fl^ mice (day 0–2) as previously described^88^ and were cultured in DMEM supplemented with 10% of heat-inactivated FBS and 2mM L-glutamine. Human astrocytes were used between passages 4 and 9. Mouse astrocytes were used between passages 2 and 6. Cells were routinely tested for *Mycoplasma* with the MycoAlert^TM^ Mycoplasma Detection Kit (Lonza) every 3–6 months at University of Pennsylvania’s Cell Center Services.

For cancer cell–astrocyte coculture experiments, astrocytes were cultured in AM until confluency and cancer cells (at 1:1 ratio with astrocytes) were added for co-culture. As control, astrocytes and cancer cells were cultured alone. Twenty-four hours after coculture, cancer cells (fluorescent-labeled) and astrocytes (non-labeled) were sorted on a BD FACSAria II for downstream analyses. The control cultured alone cancer cells and astrocytes were mixed together and then sorted. Seventy-two hours after coculture, conditioned media (CM) were collected from cocultured cells while CM from culture alone cells were mixed as control. In the indicated experiments, neutralizing antibodies targeting IFNAR1 or IFNβ were used at 2 µg/mL for 30 min prior to CM treatment.

For IFNβ treatment, murine BrM cells were treated with various concentrations of recombinant mouse IFNβ (R&D systems). Cells were harvested to test gene expression 24 h after treatment. For STAT1 and STAT3 analysis by western blot, protein lysates from human astrocytes were collected after 2 h after treatment.

The growth of cancer cells was quantified by BLI using an IVIS SpectrumCT 72 h after treatment. For IFIT1-GFP reporter cells, cells were imaged using multi-photon microscope 72 h after treatment. Gfap-Cre^+^ and Gfap-Cre^−^ *Ifnar1*^fl/fl^ mouse astrocytes were collected and purified from P0-P1 pups as described previously^88^. After obtaining a stable culture, the relative growth rates were compared using PrestoBlue resazurin assay (Thermo Fisher). PrestoBlue was added at 1:10 volumetric ratio directly to the cells in culture. After 90 min incubation, fluorescence measurements were made on a BioTek HTX plate reader. The fluorescence values were normalized to day 0 measurements made after overnight stabilization following seeding. Measurements were made every 24 h for 5 days.

### Flow cytometry and cell sorting

For single cell preparation for immune cell profiling, brain or lung metastatic lesions and lung tumor nodules were collected and incubated with 200 U/mL collagenase III (Worthington Biochem) for 20 min at 37 °C. The cell pellet was washed by resuspending in fresh MACS buffer (PBS, 0.5% BSA, 2 mM EDTA) and filtering through a 30 μm cell strainer. Spleens were collected, mechanically disaggregated, and filtered over a 70 μm cell strainer. Peripheral blood was collected by live perfusion. Red blood cells in spleen and peripheral blood samples were lysed with ACK lysing buffer.

For single cell preparation for brain cells, we followed and modified previously published procedures^89,90^. In brief, brain metastatic lesions were individually collected and digested with 0.3 U/mL Papain (Worthington Biochem), 40 U/mL DNase I (Worthington Biochem), 0.5 mM EDTA, 1 mM L-cysteine, and 0.067 mM 2-mercaptoethanol for 20 min at 37 °C. Fire polished pipettes were used to physically break down the tissues. After filtering through a 30 μm cell strainer, the cells were resuspended in 0.9 M sucrose and centrifuged at 700 × *g* for 15 min at 4 °C to remove myelin and debris. To compare astrocyte viability and numbers in Gfap-Cre^+^ and Gfap-Cre^−^ *Ifnar1*^fl/fl^ mice brains, counting beads (Thermo Fisher) were added to the stained single-cell suspension before flow cytometry analysis. For each sample, 1/2 cortex was dissociated into single-cell suspensions.

For astrocyte isolation in brain single-cell suspensions, ACSA-2 magnetic microbeads (Miltenyi Biotec) were used following the manufacturer’s instructions. In brief, cell suspension was incubated with Fc block antibody (CD16/32, 1:10 dilution) for 10 min at 4 °C, followed by incubation with ACSA-2 magnetic microbeads for 15 min at 4 °C. Labeled cells were magnetically sorted on MS columns (Miltenyi Biotec). Both the flow-through (ACSA-2^−^) and bound fractions (ACSA-2^+^) were collected for RNA extraction and flow cytometry.

For identification of immune cells or brain stromal cells, we isolate the brain metastatic lesions based on the BLI signaling. All lesions with total photon flux over 10^6^ were collected for immune cell profiling. Single-cell suspensions from brain metastatic lesions were incubated with Fc block (CD16/32, 1:10 dilution) antibody for 15 min on ice. Antibody cocktails (Supplementary Table 3) were diluted 1:100, then added and incubated for 30 min at 4 °C in the dark. Samples were washed and resuspended with fresh MACS buffer before flow cytometry analysis on a BD LSR II. All flow data were analyzed by FlowJo.

For migration assays, single cells from spleen of brain metastasis-bearing mice were stained with cell surface markers. M-MDSC and PMN-MDSC populations were sorted on a BD FACSAria II.

### Cranial chamber and intravital microscopy observation (IVM)

For intracranial window implantation, we followed previously published procedures^91,92^. In brief, mice were anesthetized with isoflurane at 1.5% (v/v) in a stereotactic device. A 4 mm in diameter circle was created on the skull and covered with 1.5 mm-thick round cover slip (Electron Microscopy Sciences). Cyanoacrylate (Vetbond Tissue Adhesive for animal use, 3 M) and a layer of dental cement were applied reinforce the window. All mice undergoing intracranial window implantation received perioperative subcutaneous administration of buprenorphine sustained release (3.25 mg/kg), dexamethasone (0.2 mg/kg) and carprofen (5 mg/kg).

For IVM in experimental mice in vivo, 5 × 10^4^ IFIT-EGFP reporter E0771-BrM cells in 100 μL of PBS injected into the left cardiac ventricle one week after intracranial window implantation. Experimental mice were kept anesthetized with 1.5% isoflurane (v/v) in a stereotactic device. To visualize the vascular structure, 100 μL of Dextran Tetramethylrhodamine 2,000,000 MW (Thermo) (10 mg/ml in PBS) was injected intravenously immediately before each imaging session. A Leica TCS SP8 MP spectral confocal and multi-photon microscope (Leica Microsystems) equipped with 2 HyD and 2 PMT detectors were used. A Coherent Chameleon XR femto-second pulsed laser (Coherent, Inc.) was used at 940 nm and images were acquired using a ×25 1.0 water immersion objective with an 8000 Hz resonant scanner. For each image, stack laser intensity was adjusted according to imaging depth to maximize image intensity and minimize saturation throughout the stack acquisition. A Leica ×10 0.04NA objective and Nikon Fi3 3-color camera were used to capture low magnification, reflected light, stitched color images of the overall window area. Images were deconvoluted using Huygens (Scientific Volume Imaging B.V.) and ImageJ software. Deconvolution was applied for minimizing noise and artifacts caused by breathing of the animal and performed using the same settings for all images. Analysis was performed on deconvoluted raw data, but all presented images are pseudo-colored and contrast enhanced for clarity. For IVM in IFNβ-treated cells in vitro, images were acquired at single stacks per field, using the same laser configurations and objective used for in vivo imaging.

IFIT-GFP intensities were analyzed and quantified by ImageJ. For in vivo images, we used deconvoluted image stacks acquired at 512 μm × 512 μm and optical sectioning at every 5 μm. Cells located within blood vessels were not counted by 3D visualization to ensure that counted cell bodies were not inside blood vessels. For in vitro images, raw data were analyzed, but the presented images are pseudo-colored and contrast enhanced for clarity. Care was taken to apply the same settings for all images. For fluorescence intensity quantification of all in vivo and in vitro images, images at 8-bit resolution were used for generating binary images, allowed the creation of ROI for each cell and for background subtraction. Each image’s ROI mask was then applied individually to both GFP and tdTomato channels, allowing collection of total fluorescence intensity measurements for each cell. Background values were subtracted from the values obtained from each cell and graphs were plotted with the total fluorescence intensity ratio between GFP and tdTomato.

### tdTomato staining and quantification

Whole mouse brains were fixed in 4% PFA overnight at 4 °C. Then, brains were washed with PBS and incubated for an additional 24 h in PBS. Finally, brains were sequentially incubated overnight in 15% and 30% sucrose solutions prepared in PBS. 80 µm coronal slices were cut throughout the whole brain using a cryotome, and all brain slices were evenly distributed into 10 collection tube containing anti-freeze media (30% PEG300 and 30% glycerol in PBS). The sections were stored at −20 °C until use.

For staining, 1 set of free floating sections (1/10 of the whole brain) were placed into a dish and washed three times with PBS containing 0.25% Triton-X100 (PBST). Sections were blocked and permeabilized for 1 h at room temperature using PBST containing 2% BSA and 5% donkey serum. The tdTomato primary antibody (OriGene) was prepared at 1:250 dilution in this buffer and incubated with the sections overnight at 4 °C. The next day, sections were washed with PBST three times and incubated for 2 h in the dark with secondary antibody diluted 1:500 in PBST. Nuclei were counterstained by incubating with 2 µM DAPI for 15 min at room temperature. Sections were mounted on glass slides and coverslips using VECTASHIELD mounting medium. Slides were scanned at ×20 magnification using a Nikon ECLIPSE Ni-E microscope. Analysis was performed using the NIS-Elements software (Nikon). Total tissue area in each scan were automatically detected and drawn. Lesions areas were manually drawn in the software based on the tdTomato staining. Singlets were defined by delocalized cells having tdTomato^+^ staining. Using the area of an identified single cell, micrometastases were defined by clusters of 10–100 cells by area and macrometastases as >100 cells by area.

### Migration assay

Ninety-six-well disposable chemotaxis plates (Neuro Probe, SKU: 101-5) were used to set up the immune cell migration assays. M-MDSC and PMN-MDSC sorted from spleens of brain metastasis-bearing mice are loaded. Two to 5 × 0^4^ sorted immune cells (M-MDSC or PMN-MDSC) were added into the top compartments. In the bottom compartment, conditioned media (CM) were added. CM from cocultured and cultured alone cancer cells were collected 48 h after seeding the cells, centrifuged, and filtered through a 0.22 µm membrane. In the indicated experiments, CM were pretreated with 2 µg/mL neutralizing mouse CCL2 antibody or the matched goat IgG isotype control (Novus Biologicals) for 30 min at 37 °C. The assembled chamber was incubated at 37 °C for 3 h. Cells that migrated into the bottom compartment were fixed by directly adding 16% paraformaldehyde (final concentration 4%) containing Hoechst 33342 to stain nuclei. The plate was scanned and nuclei counting analysis was performed using the Nikon Elements AR software.

### RNA extraction, RT-PCR, and western blot

For cells sorted from co-culture experiments, total RNA was extracted using the Direct-Zol^RM^ RNA MiniPrep Plus (Zymo Research) according to the manufacturer’s protocol. For isolated cells from experimental mice, RNA was extracted using the Quick-RNA microprep kit (Zymo Research) according to the manufacturer’s protocol. Reverse transcription was carried out using the RevertAid RT kit (Thermo Fisher) following manufacturer’s protocol. Primer sequences used are listed in Supplementary Tables 1 and 2. Reactions were performed using Powerup SYBR Green Master Mix (Applied Biosystems) using QuantStudio 6 Flex. Relative gene expression was calculated by ddCT method normalized to β-actin on the QuantStudio Real-Time PCR Software v.1.2 (Applied Biosystems).

For western blot, total protein lysate was extracted from astrocytes using RIPA lysis buffer containing protease (Roche) and phosphatase inhibitors (Thermo Fisher). Proteins were separated by SDS-PAGE and transferred to nitrocellulose membranes (Bio-Rad). Membranes were washed and blocked with Odyssey blocking buffer (Li-Cor) and stained for primary antibodies overnight at 4 °C. The membranes were washed and probed with secondary antibodies for 2 h in the dark at room temperature. Blots were scanned using an infrared scanner (Li-Cor).

### RNA-seq, IPA analysis, and bioinformatic analyses of clinical datasets

mRNA purified from cancer cells (*n* = 3 biologically independent experiments) was used. Sequencing libraries were prepared from RNA samples using QuantSeq (Lexogen). RNA-seq data was aligned using bowtie2^93^ algorithm against hg38 human genome version and RSEM v1.2.12 software^94^ was used to estimate read counts and RPKM values using gene information from Ensemble transcriptome version GRCh38.p12. Raw counts were used to estimate significance of differential expression difference between two experimental groups using DESeq2^95^. Overall gene expression changes were considered significant if passed FDR < 5% thresholds unless stated otherwise. Gene set enrichment analysis was done using QIAGEN’s Ingenuity® Pathway Analysis software (IPA®, QIAGEN, www.qiagen.com/ingenuity) using “Canonical pathways” options. Enriched pathways that passed FDR < 5% threshold and were predicted to be activated based on positive predicted activation state *Z*-score were reported. All these were performed by Wistar’s Genomics and Bioinformatics Facilities.

For clinical validations, we analyzed 2 breast cancer cohorts with paired primary and brain metastatic tumor samples (PMC6449168 and GSE125989)^49,50^, another breast cancer dataset with unpaired brain and lung metastatic samples (GSE14020)^51,52^, and a melanoma dataset containing paired and unpaired brain metastatic and extracranial tumors (EGAD00001005046)^53^. Bioinformatic tools were used including CIBERSORT, MCP-counter and xCell^54–56^. Total immune scores were generated by bioinformatic tools including CIBERSORT and xCell. In MCP-counter analyses, monocytic lineage, neutrophil, T cell, B cell, and NK cell scores were used for further analyses on indicated subpopulations. In CIBERSORT analyses, monocytic myeloid cell scores were generated by adding monocyte, macrophage M0, macrophage M1 and macrophage M2 scores. Lymphoid cell scores were generated by adding naive B cell, memory B cell, plasma cell, CD8 T cell, naive CD4 T cell, memory resting CD4 cell, memory activated CD4 cell, follicular helper T cell, regulatory T cell and gamma/delta T cell scores. T lymphoid cell scores include CD8 T cell, naive CD4 T cell, memory resting CD4 cell, memory activated CD4 cell, follicular helper T cell, regulatory T cell and gamma/delta T cell scores. B lymphoid scores include naive B cell, memory B cell and plasma cell scores. NK cell scores were generated by adding resting NK cell and activated NK cell scores. Correlations between CCL2 expression and immune scores were analyzed using Simple Linear Regression in Prism software. We used online bioinformatic tool^45^ to analyze chemokine receptor expression in immune cells in clinical samples of brain metastasis.

Single-cell RNAseq analysis of clinical samples was performed in R using the Seurat (v4.1) package^96^. A Seurat object was generated using filtered matrices, gene names, and bar codes downloaded for each dataset (GSE174401^58^, GSE176078^59^, GSE186344^57^). Individual samples from each dataset were assigned a metadata identifier as either brain metastasis or extracranial tumor and processed for quality control. Apoptotic and non-viable cells were removed by excluding any cells with mitochondrial transcripts >20%. Empty vectors and multiplets were removed by excluding cells in the top and bottom 0.5% of the unique molecular identifier (UMI) distribution. For each dataset, the filtered samples were merged for normalization, principal component (PC) analysis, and unsupervised clustering. For clustering, we used the first 30 PCs to visualize clusters by uniform manifold approximation and projection (UMAP). We used the first 5 PCs to construct the shared nearest neighbor graph and used a resolution of 0.5 to optimize the modularity function and define clusters. Cell-type annotations for each cluster was conducted manually using published gene signatures for myeloid cells^97^, T cells^98^, NK cells^99^, and B cells^100^. *PTPRC* (CD45 gene) was used as a pan-immune cell marker. Myeloid clusters were identified by expression of *LYZ*, *CD14*, *GCGR3A*, *MRC1*, and *AIF1*. T/NK clusters were identified by the expression of *TRBC2*, *CD3E*, *IL7R*, and *KLRB1*. Some of the T cell clusters also contained exhaustion markers such as *TOX* and *LAG3*. B cells were identified by expression of *CD19*, *MS4A1*, and *CD79A*, and differentiated plasma cells were further identified by expression of *XBP1* and *MZB1*. We further calculated the numbers of myeloid cells (adding all identified myeloid clusters), lymphoid cells (add all identified T/NK, T, B, and plasma cell clusters) and total immune cells (adding all myeloid and lymphoid cells). The proportion of total immune, myeloid cells and lymphoid cells were calculated out of total cells in each sample.

### Reporting summary

Further information on research design is available in the Nature Portfolio Reporting Summary linked to this article.

## Supplementary information

Supplementary Information

Reporting Summary

## Data Availability

The RNAseq data generated in this study have been deposited in the NCBI GEO database under accession code GSE224302. Single-cell RNAseq data used for meta-analysis are available from the NCBI GEO database under accession codes GSE174401, GSE176078, and GSE186344. Bulk RNAseq data used for meta-analysis are available from the NCBI GEO database under accession codes GSE125989 and GSE14020. Additional bulk RNAseq data were obtained after requesting access (EGAD00001005046 and PMC6449168) from the corresponding authors of those studies. The remaining data are available within the article, Supplementary Information, or Source data file. Source data are provided with this paper.

## Source data

Source Data
