# Supplementary material for: Type I interferon response in astrocytes promotes brain metastasis by enhancing monocytic myeloid cell recruitment
Source: Nat Commun. 2023 May 6;14:2632. doi: 10.1038/s41467-023-38252-8 (PMC10163863; doi:10.1038/s41467-023-38252-8)
Supplement: Supplementary file 1 — Supplementary Information [file 41467_2023_38252_MOESM1_ESM.pdf]

SUPPLEMENTARY INFORMATION

Type I Interferon Response in Astrocytes Promotes Brain Metastasis by Enhancing Monocytic Myeloid Cell Recruitment

Weili Ma[1*], Maria Cecília Oliveira-Nunes[1,6*], Ke Xu[2], Andrew Kossenkov[3], Benjamin C. Reiner[4], Richard C. Crist[4], James Hayden[5], Qing Chen[1]

[1] Immunology, Microenvironment and Metastasis Program, The Wistar Institute, Philadelphia, PA 19104, USA

[2] MD/PhD Program, Boston University School of Medicine, Boston, MA 02215, USA

[3] Gene Expression & Regulation Program, The Wistar Institute, Philadelphia, PA 19104, USA

[4] Department of Psychiatry, Perelman School of Medicine, The University of Pennsylvania, Philadelphia, PA 19104, USA

[5] Imaging Shared Resource, The Wistar Institute, Philadelphia, PA 19104, USA

[6] Current Address: Carisma Therapeutics, Philadelphia, PA 19104, USA

* These authors contributed equally

Correspondence: qichen@wistar.org (Qing Chen)

Supplementary Figures 1–6

Supplementary Tables 1-4

Supplementary Fig. 1

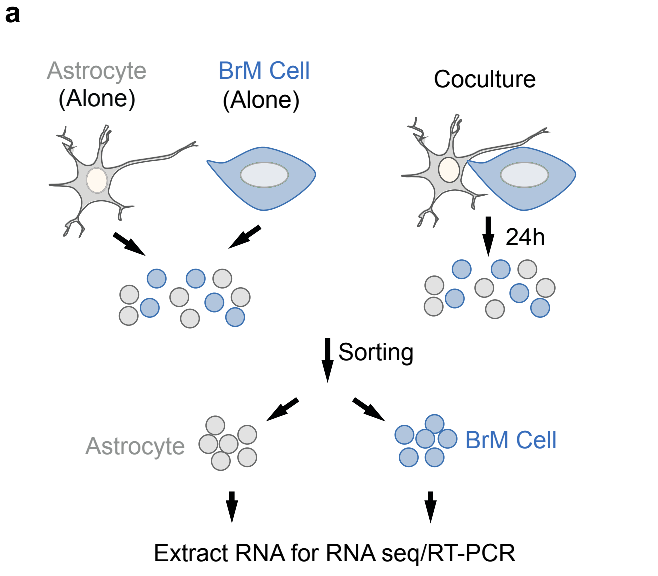

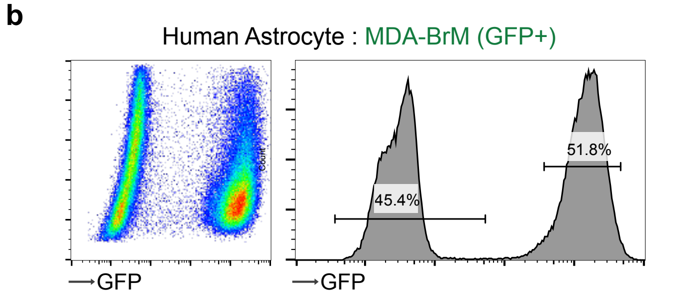

c

Breast Cancer	Source	Stable Labels
MDA231-BrM	Human (female)	Luciferase, GFP
E0771-BrM	Mouse (female)	Luciferase, tdTomato
A7C11-BrM	Mouse (female)	Luciferase, tdTomato
**Melanoma**	**Source**	**Stable Labels**
Yumm 1.7-BrM	Mouse (male)	Luciferase, tdTomato

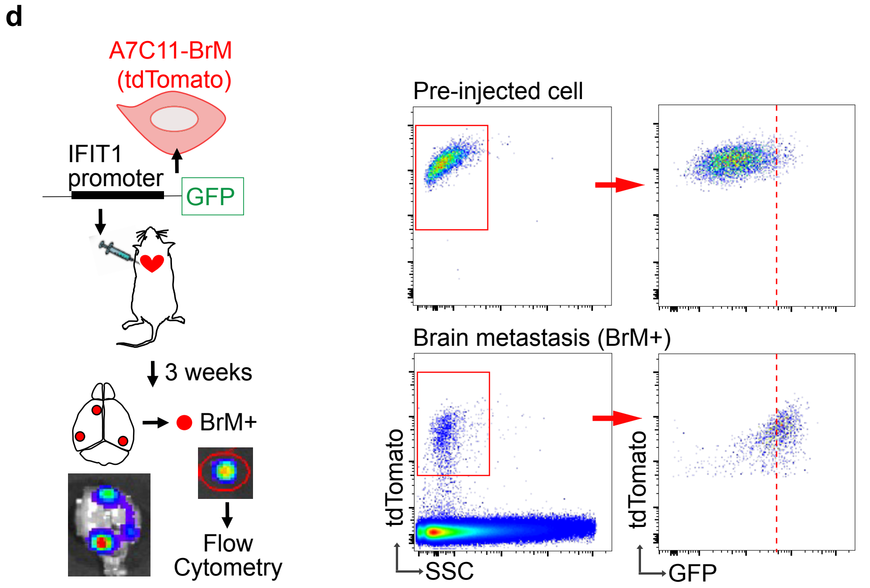

Supplementary Fig. 1 IFN signaling is activated in brain metastasis.

a,**b**, *In vitro* astrocyte-BrM coculture experiments. (**a**) Scheme of experiment setup. (**b**) Representative flow cytometry profiles of sorted astrocytes and BrM cells.

c, BrM cells used for *in vitro* coculture and *in vivo* brain metastasis experiments, including newly established E0771-BrM, A7C11-BrM and previously established MDA231-BrM and Yumm1.7-BrM.

d, IFIT1-GFP reporter structure is expressed in A7C11-BrM cells. 3 weeks after injecting the reporter BrM cells, macrometastatic lesions are isolated based on the luciferase signals in bioluminescent images (BLI), defined as BrM+ tissues. GFP expression in tdTomato$^+$ BrM cells is detected by flow cytometry.

Supplementary Fig. 2

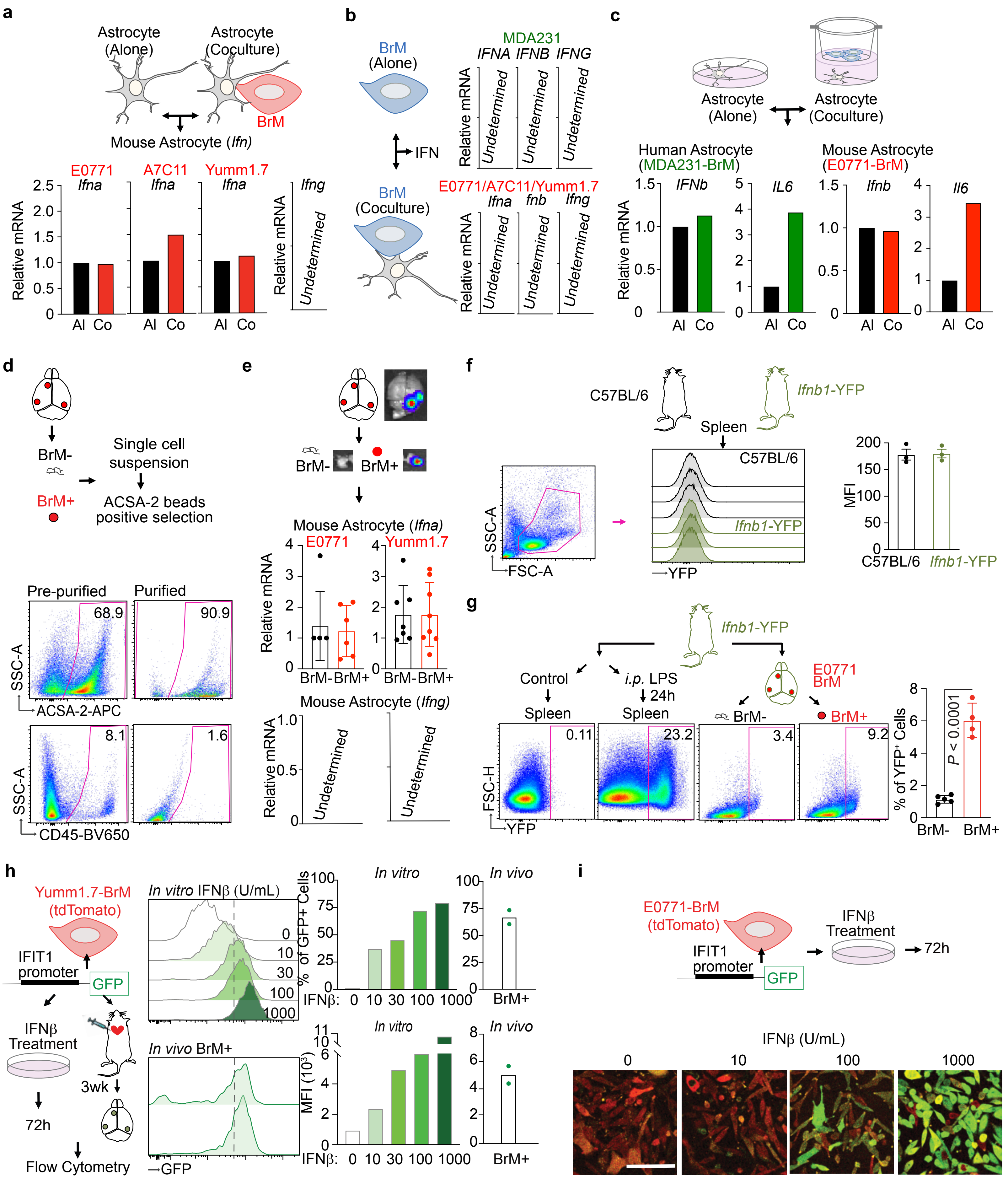

Supplementary Fig. 2 Low level of type I IFN signaling in brain metastasis microenvironment.

a, No change of IFNα in mouse astrocytes after cocultured with BrM cells *in vitro*. No detectable IFNγ expression in mouse astrocytes. RT-PCR results of *Ifna* and *Ifng* genes. Representative data shown from 3 biologically independent experiments for each BrM cell.

b, No detectable IFN cytokines in human or mouse BrM cells by RT-PCR.

c, Contact-independent coculture of astrocyte and BrM cells. RT-PCR results of the expression of IFNβ and IL-6 the astrocytes cocultured with BrM cells. Representative data shown from 3 biologically independent experiments for each BrM cell.

d, Astrocyte isolation. Representative results from one astrocyte purification experiment using a macrometastatic lesion established by E0771-BrM cells. ACSA-2 and CD45 are detected by flow cytometry to measure the percentage of astrocytes and immune cells, respectively, before and after purification.

e, IFNα and IFNγ production in metastasis-activated astrocytes *in vivo*. From the experimental mice developed with brain metastasis, metastatic lesions (BrM+) and metastasis-free tissues (BrM-) are isolated based on the luciferase signals in BLI. Astrocytes are purified by ACSA-2 magnetic beads and *Ifna* and *Ifng* expression is detected by RT-PCR. Data are from merged samples from at least 3 biologically independent experiments and presented as mean ± S.D. E0771 model (BrM-: n = 4, BrM+: n = 6). Yumm1.7 model (BrM-: n = 7, BrM+: n = 8). Source data are provided as a Source Data file.

f, Basal YFP level in *Ifnb1*-YFP reporter mice. YFP signal in the splenocytes from wild type (WT) and *Ifnb1*-YFP are detected and quantified by flow cytometry. n = 3 mice. Data are presented as mean ± S.D. Source data are provided as a Source Data file.

g, Defining YFP^+ cells in *Ifnb1*-YFP reporter mice. Splenocytes from control and LPS treated mice are used to gate YFP^+ cells. From the experimental mice with developed brain metastasis, metastatic lesions (BrM+) and metastasis-free tissues (BrM-) are isolated based on the luciferase signals in BLI. Percentages of YFP^+ cells out of total live cells are shown in the bar graph. Data are presented as mean ± S.D. (BrM-: n = 5, BrM+: n = 4). *P* value is the result from unpaired two-tailed t test. Source data are provided as a Source Data file.

h, IFIT1-GFP reporter structure is expressed in Yumm1.7-BrM cells. The reporter cells treated with various concentrations of IFNβ *in vitro* and GFP expression is quantified. 3 weeks after injecting the reporter BrM cells, macrometastatic lesions are isolated for flow cytometry analyses. The GFP expression in $tdTomato^+$ BrM cells *in vivo* is quantified and compared to the IFNβ-treated cells. The percentage of GFP^+ cells and mean fluorescent intensity (MFI) are quantified in $tdTomato^+$ BrM cells. Data are from merged samples from 2 biologically independent experiments. Source data are provided as a Source Data file.

i, Representative images of IFNβ-treated IFIT1-GFP expressing E0771-BrM reporter cells observed by IVM. Scale bar, 100 μm. Representative data shown from 3 biologically independent experiments.

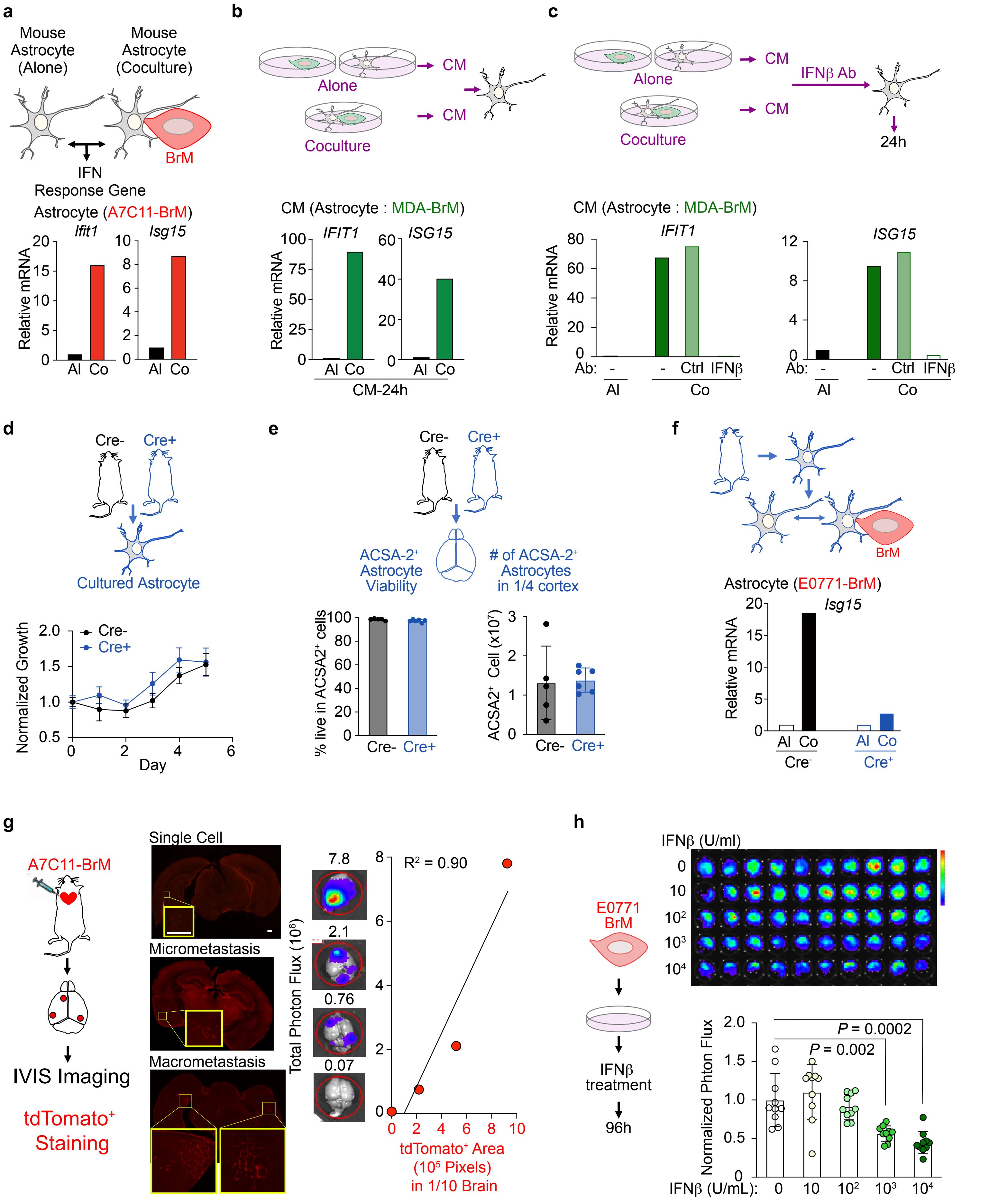
a
Mouse Astrocyte (Alone)
Mouse Astrocyte (Coculture)
BrM
IFN Response Gene
Astrocyte (A7C11-BrM)
Ifit1
Isg15
Relative mRNA
Al Co
b
Alone
Coculture
CM
CM (Astrocyte : MDA-BrM)
IFIT1
ISG15
CM-24h
c
IFNβ Ab
24h
Ab: - - Ctrl IFNβ
Al
Co
d
Cre-
Cre+
Cultured Astrocyte
Normalized Growth
Day
e
ACSA-2+ Astrocyte Viability
# of ACSA-2+ Astrocytes in 1/4 cortex
% live in ACSA2+ cells
ACSA2+ Cell (x10^7)
f
Astrocyte (E0771-BrM)
g
A7C11-BrM
IVIS Imaging
tdTomato+ Staining
Single Cell
Micrometastasis
Macrometastasis
Total Photon Flux (10^6)
R² = 0.90
tdTomato+ Area (10^5 Pixels) in 1/10 Brain
h
IFNβ (U/ml)
E0771 BrM
IFNβ treatment
96h
Normalized Phton Flux
P = 0.0002
P = 0.002
IFNβ (U/mL): 0 10 10² 10³ 10⁴

Supplementary Fig. 3 Type I IFN activation in astrocytes promotes brain metastasis.

a, Increased IFN response genes in mouse astrocytes cocultured with breast cancer A7C11-BrM *in vitro*. The expression of IFN response genes are measured by RT-PCR. Al, cultured alone astrocytes; Co, cocultured astrocytes. Representative data shown from 3 biologically independent experiments for each BrM cell.

b,c, Human astrocytes are treated with conditioned media (CM) and type I IFN response genes are detected by RT-PCR. (**b**) CM are collected from astrocyte-BrM coculture (Co) or cultured alone (AL). Representative data shown from 3 biologically independent experiments for each BrM cell. (**c**) CM are pretreated with either neutralizing antibody against IFNβ or the matched IgG control antibody (Ctrl). Representative data shown from 3 biologically independent experiments for each BrM cell.

d,e, Characterization of astrocytes from astrocyte-specific IFNAR1 knock out mice. Cre-, *Gfap*-Cre$^{-/-}$; *Ifnar1*$^{f/f}$ mice; Cre+, *Gfap*-Cre$^{+/-}$; *Ifnar1*$^{f/f}$ mice. (**d**) Growth of primary cultured astrocytes. n = 10 samples in each condition. 2 biologically independent experiments were conducted. Data are presented as mean ± S.D. Source data are provided as a Source Data file. (**e**) Viabilities and numbers of astrocytes isolated from mouse brain. 1/4 of cortex of each mouse are collected. Data are from merged samples from 2 biologically independent experiments presented as mean ± S.D. (Cre-: n = 6, Cre+: n = 5). Source data are provided as a Source Data file.

f, RT-PCR results of the type I IFN response gene, *Isg15*, in the astrocytes coculture with BrM cells *in vitro*. Primary cultured astrocytes are isolated from transgenic mice. Cre-, *Gfap*-Cre$^{-/-}$; *Ifnar1*$^{f/f}$ mice; Cre+, *Gfap*-Cre$^{+/-}$; *Ifnar1*$^{f/f}$ mice. Representative data shown from 3 biologically independent experiments for each BrM cell. Source data are provided as a Source Data file.

g, Correlation between the total photon flux from IVIS imaging and immunofluorescence analyses of the brain metastatic regions in 1/10 of whole brain sections. Brain metastatic regions are defined based on tdTomato staining. Representative images of single metastatic cells, micrometastasis and macrometastasis. Bar = 400μm. Representative data shown from 2 biologically independent experiments. Source data are provided as a Source Data file.

h, E0771-BrM cells are treated with various doses of recombinant mouse IFNβ and cell growth is quantified by BLI after 72 hours. 2 biologically independent experiments were performed with similar results (n = 10 per concentration in each experiment). Data are presented as mean ± S.D. *P* values are the results from paired two-tailed t test. Source data are provided as a Source Data file.

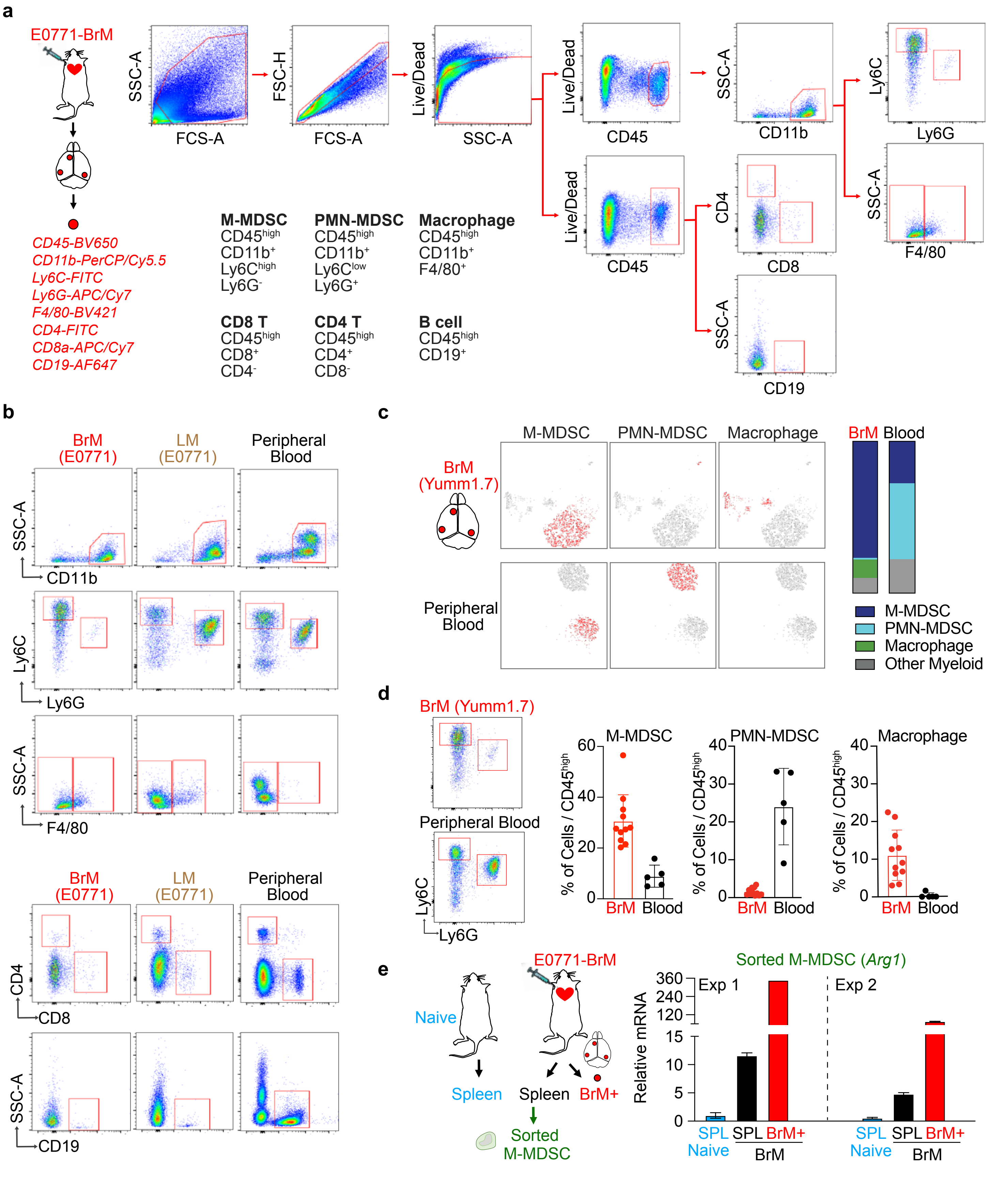
a
E0771-BrM
CD45-BV650
CD11b-PerCP/Cy5.5
Ly6C-FITC
Ly6G-APC/Cy7
F4/80-BV421
CD4-FITC
CD8a-APC/Cy7
CD19-AF647
SSC-A
FCS-A
FSC-H
Live/Dead
CD45
CD11b
Ly6C
Ly6G
CD4
CD8
F4/80
CD19
M-MDSC
CD45high
CD11b+
Ly6Chigh
Ly6G-
PMN-MDSC
Ly6Clow
Ly6G+
Macrophage
F4/80+
CD8 T
CD8+
CD4-
CD4 T
CD4+
CD8-
B cell
CD19+
b
BrM (E0771)
LM (E0771)
Peripheral Blood
c
BrM (Yumm1.7)
BrM
Blood
Other Myeloid
d
% of Cells / CD45high
e
Naive
Spleen
BrM+
Sorted M-MDSC
Sorted M-MDSC (Arg1)
Relative mRNA
Exp 1
Exp 2
SPL

Supplementary Fig. 4 Immune cell profiles in metastatic lesions.

a, Representative flow cytometry profiles of immune cells in one brain metastatic lesions established by E0771-BrM cells. M-MDSC, monocytic myeloid-derived suppressor cells; PMN-MDSC, polymorphonuclear MDSC; CD8 T, CD8 T lymphocyte; CD4 T, CD4 T lymphocyte; B cell, B lymphocyte.

b, E0771-BrM cell are injected into experimental mice to establish brain or lung metastasis. Representative dot plots of myeloid and lymphoid subpopulations of one brain metastatic lesion, merged lung metastatic samples from one experimental mouse and the blood sample from one brain metastasis-bearing mouse.

c,**d**, Yumm1.7-BrM cells are injected into experimental mice to establish brain metastasis. (**c**) Representative tSNE files of myeloid subpopulations of one brain metastatic lesion and the blood sample from one brain metastasis-bearing mouse. Bar graphs indicate the proportions of indicated immune subpopulations in $CD45^{high}$ $CD11b^{+}$ myeloid cells (total is 100%). (**d**) Dot plots show the representative flow profiles of MDSCs. Bar graphs show the percentages of myeloid subpopulations out of $CD45^{high}$ cells. Data are from merged samples of 2 biologically independent experiments and presented as mean ± S.D. (BrM: n = 11, blood: n = 5). Source data are provided as a Source Data file.

e, RT-PCR results of immunosuppressive gene, *Arg1*, in the sorted M-MDSC from naïve and brain metastasis-bearing mice. SPL, spleen; BrM, brain metastatic lesions. Representative data shown from 3 biologically independent experiments.

Supplementary Fig. 5

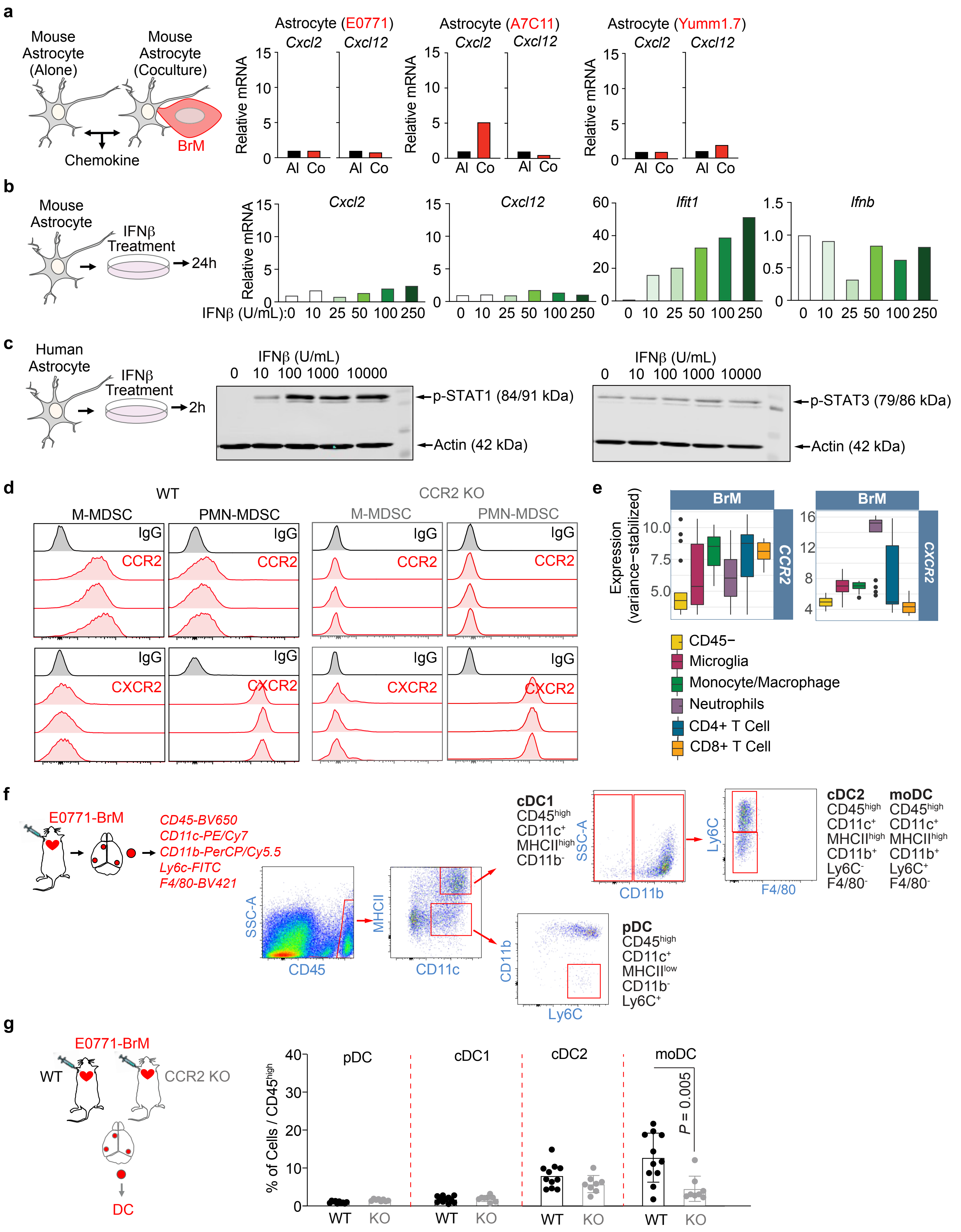

Supplementary Fig. 5 CCR2 mediates M-MDSC migration in brain metastasis.

a, RT-PCR results of the expression of chemokines in astrocytes cocultured with BrM cells *in vitro*. Mouse astrocytes are cocultured with breast cancer E0771-BrM, A7C11-BrM and melanoma Yumm1.7-BrM. The expressions of CXCL2 and CXCL12 are measured. Al, cultured alone astrocytes; Co, cocultured astrocytes. Representative data shown from 3 biologically independent experiments for each BrM cell.

b, RT-PCR results of the expression of chemokines, IFIT1 and IFNβ-treated astrocytes *in vitro*. Mouse astrocytes cells are treated with various concentrations of IFNβ and the gene expressions. *Ifit* is used as a positive control for IFN responses. Representative data shown from 3 biologically independent experiments.

c, Western blot results of phosphorylated STAT1 (p-STAT1) and phosphorylated STAT3 (p-STAT3) in IFNβ-treated astrocytes *in vitro*. 2 biologically independent experiments were conducted with similar results. Source data are provided as a Source Data file.

d, CCR2 expression in monocytic myeloid cells. E0771-BrM cells are injected into wild type (WT) or CCR2 knock out (KO) mice. Spleens are harvested and the chemokine receptors in myeloid subpopulations are detected by flow cytometry. M-MDSC, monocytic myeloid-derived suppressor cells; PMN-MDSC, polymorphonuclear MDSC. Representative data from 3 biologically independent experiments.

e, Bioinformatic analyses of chemokine receptors in myeloid immune subpopulations from 89 clinical brain metastasis samples (https://joycelab.shinyapps.io/braintime/).

f, Representative flow cytometry profiles of dendritic cells (DC) in one brain metastatic lesions established by E0771-BrM cells. pDC, plasmacytoid DC; cDC1, conventional type 1 DC; cDC2, conventional type 2 DC; moDC, monocyte-derived DC.

g, Percentages of DC subpopulations out of $CD45^{high}$ cells in brain metastatic lesions. Data are merged samples from n = 3 biologically independent experiments and presented as mean ± S.D. *P* values are the results from unpaired two-tailed t test. (pDC: n = 10 WT, n = 8 CCR2KO. cDC1, cDC2, moDC: n = 11 WT, n = 8 CCR2KO). Source data are provided as a Source Data file.

Supplementary Fig. 6

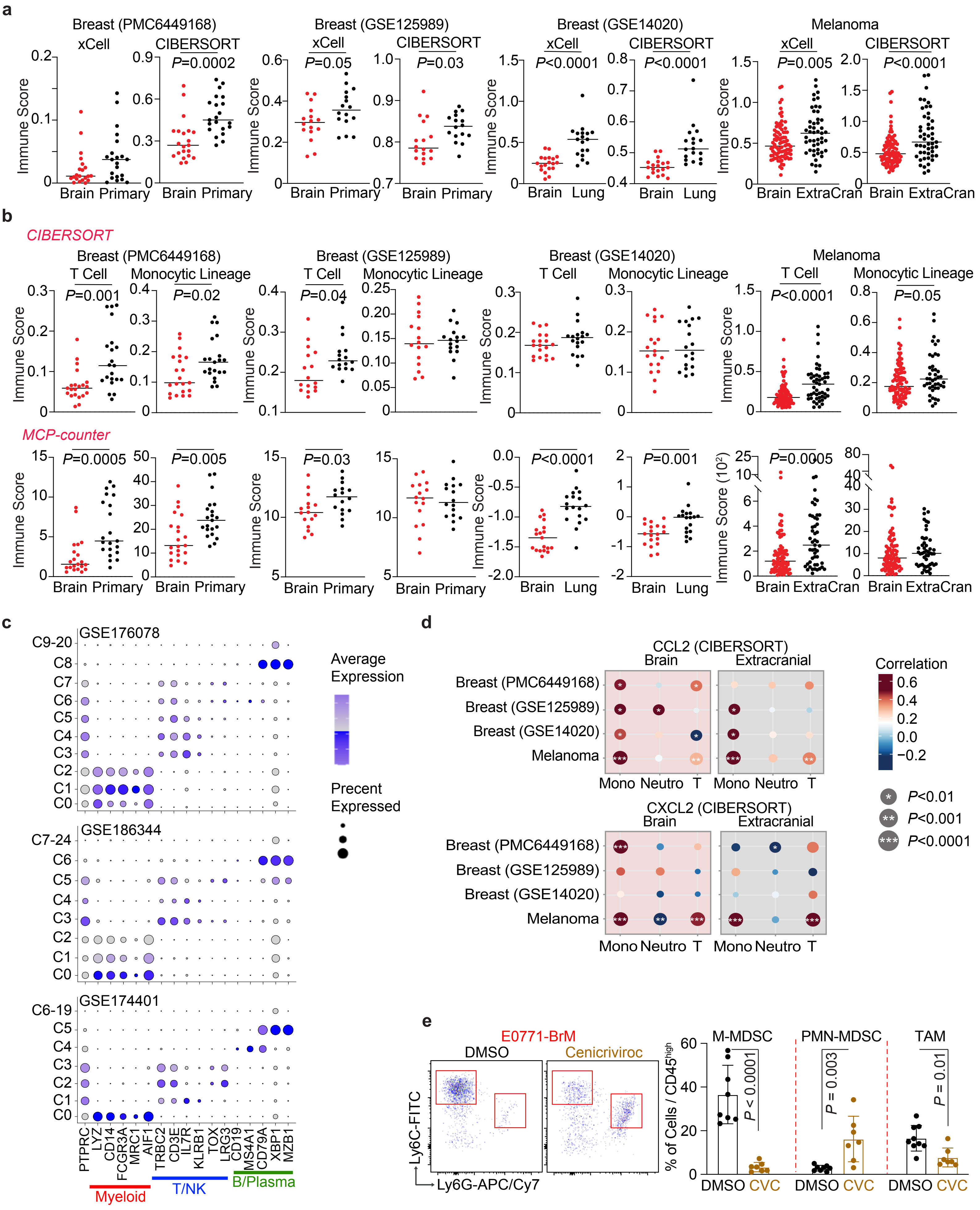

Supplementary Fig. 6 Correlation of CCL2 expression and monocytic myeloid immune cells score in clinical brain metastasis samples.

a,b, Bioinformatic analyses of immune cells in clinical samples. We include breast cancer PMC6449168 and GSE125989 (paired primary and brain metastatic datasets), GSE14020 (unpaired brain and lung metastatic samples), and melanoma EGAD00001005046 (paired and unpaired brain metastatic and extracranial tumors) in the analyses. (**a**) Total immune scores in brain metastatic (Brain), extracranial (ExtraCran) lung metastatic (Lung) or primary tumors analyzed by CIBERSORT and xCell. Source data are provided as a Source Data file. (**b**) Immune scores of and T cell and monocytic lineage subpopulations are analyzed by CIBERSORT and MCP-counter. *P* values are the results from unpaired two-tailed t test. PMC6449168: n = 22. GSE125989: n = 16. GSE14020: n = 19 Brain, n = 18 Lung. MD Melanoma: n = 88 Brain, n = 49 Extracranial. Source data are provided as a Source Data file.

c, Dot plots of representative genes used for single cell RNAseq cluster annotations in each dataset. Pan-immune cells (*PTPRC*). Myeloids (*LYZ, CD14, GCGR3A, MRC1, AIF1*). T/NK (*TRBC2, CD3E, IL7R, KLRB1*, *TOX*, *LAG3*). B/Plasma cells (*CD19, MS4A1, CD79A*, *XBP1, MZB1*).

d, Correlations of CCL2 or CXCL12 expression and immune scores of monocytic lineage (Mono), neutrophil (Neutro) and T lymphoid (T) subpopulations in brain metastatic and extracranial tumors analyzed by CIBERSORT. Breast cancer PMC6449168 and GSE125989 (paired primary and brain metastatic datasets), GSE14020 (unpaired brain and lung metastatic samples), and melanoma EGAD00001005046 (paired and unpaired brain metastatic and extracranial tumors) are analyzed. *P* values are the results from unpaired two-tailed t test. Source data are provided as a Source Data file.

e, CCR2/CCR5 antagonist treatment blocks monocytic myeloid cell infiltration. Bar graphs show the percentages of myeloid subpopulations out of $CD45^{high}$ cells. M-MDSC, monocytic myeloid-derived suppressor cells; PMN-MDSC, polymorphonuclear MDSC. Data are merged samples from 2 biologically independent experiments and presented as mean ± S.D (M-MDSC, PMN-MDSC: n = 8 DMSO, n = 7 CEN. TAM: n = 9 DMSO, n = 7 CEN). *P* values are the results from unpaired two-tailed t test. Source data are provided as a Source Data file.

Supplementary Table 1: List of Primers used for RT-PCR (mouse genes)

Gene	Sense (5'-3')	Antisense (3'-5')	Acession No.
Ccl2 (set1)	CCCACTCACCTGCTGCTACT	TCTGGACCCATTCCTTCTTG	NM_011333
Ccl2 (set2)	TCCCAATGAGTAGGCTGGAG	GCTGAAGACCTTAGGGCAGA	NM_011333
Ifng	GCCACGGCACAGTCATTGA	TGCTGATGGCCTGATTGTCTT	NM_008337
Cxcl12	TGCATCAGTGACGGTAAACCA	CACAGTTTGGAGTGTTGAGGAT	NM_001012477
Actb	GGCTGTATTCCCCTCCATCG	CCAGTTGGTAACAATGCCATGT	NM_007393
Cxcl2	AGTGAACTGCGCTGTCAATG	CTTCAGGGTCAAGGCAAACT	NC_000071.6
Cxcl3	CTCCAGACTCCAGCCACACT	GTCACCGTCAAGCTCTGGAT	NC_000071
Cxcl5	TGCCCTACGGTGGAAGTCATA	TGCATTCCGCTTAGCTTTCTTT	NM_009141
Ifit1	CTGAGATGTCACTTCACATGGAA	GTGCATCCCCAATGGGTTCT	NM_008331
Ifnar1	GGTGGTTCTGTCTCGGTGTT	GCCAGCTCCTCCAGTTAGTG	NM_010508
Ifnb1	CAGCTCCAAGAAAGGACGAAC	GGCAGTGTAACTCTTCTGCAT	NM_010510
Isg15	GGTGTCCGTGACTAACTCCAT	TGGAAAGGGTAAGACCGTCCT	NM_015783
Usp18	TTGGGCTCCTGAGGAAACC	CGATGTTGTGTAAACCAACCAGA	NM_011909
B2m	GGCCCATCTTGCATTCTAGGG	CAGGCAACGGCTCTATATTGAAG	NM_009735

Supplementary Table 2: List of Primers used for RT-PCR (human genes)

Gene	Sense (5'-3')	Antisense (3'-5')	Acession No.
ACTB	AAACTGGAACGGTGAAGGTG	AGAGAAGTGGGGTGGCTTTT	NM_001101.3
B2M	CTCCGTGGCCTTAGCTGTG	TTTGGAGTACGCTGGATAGCCT	NM_004048
CCL2	CCCCAGTCACCTGCTGTTAT	TCCTGAACCCACTTCTGCTT	NM_002982
CXCL12	CTACAGATGCCCATGCCGAT	CAGCCGGGCTACAATCTGAA	NM_001178134
CXCL2	GAAAGCTTGTCTCAACCCCG	GTTGGATTTGCCATTTTTCAGCA	NC_000004
IFIT1	GCCCAGACTTACCTGGACAA	GGTTTTCAGGGTCCACTTCA	NM_001270930
IFNA1	CTGTGTGATGCAGGAGGAGA	GATCTCATGATTTCTGCTCTGA	NM_024013
IFNAR1	AACAGGAGCGATGAGTCTGTC	TGCGAAATGGTGTAAATGAGTCA	NM_000629
IFNB1	ATGACCAACAAGTGTCTCCTCC	GGAATCCAAGCAAGTTGTAGCTC	NM_002176
IFNG1	TCGGTAACTGACTTGAATGTCCA	TCGCTTCCCTGTTTTAGCTGC	NM_000619
ISG15	TGTCGGTGTCAGAGCTGAAG	GCCCTTGTTATTCCTCACCA	NM_005101
USP18	CAGACCCTGACAATCCACCT	AGCTCATACTGCCCTCCAGA	NM_017414

Supplementary Table 3: List of Antibodies

	Company	Catalog#	Clone	Application
CD16/CD32	BD Biosciences	553142	2.4G2	Fc blocking antibody
CD45-BV650	BD Biosciences	563410	30-F11	Flow cytometry
Ly6G-APC/Cy7	BioLegend	127623	1A8	Flow cytometry
CD11b-PerCP/Cy5.5	BioLegend	101227	M1/70	Flow cytometry
Ly6C-FITC	BD Biosciences	561085	Al-21	Flow cytometry
F4/80-BV421	BioLegend	123131	BM8	Flow cytometry
CD11c-PE/Cy7	BioLegend	117318	N418	Flow Cytomtery
I-A/I-E (MHCII)-APC	BioLegend	117613	M5/114.15.2	Flow Cytometry
CD4-FITC	BioLegend	100405	GK1.5	Flow cytometry
CD8a-APC/Cy7	BioLegend	100713	53-6.7	Flow cytometry
CD19-AF647	BioLegend	115522	6D5	Flow cytometry
CCR2-AF647	BioLegend	150603	SA203G11	Flow cytometry
CXCR2-AF647	BioLegend	149305	SA044G4	Flow cytometry
Rat IgG2b-AF647	BioLegend	400626	RTK4530	Isotype control for CCR2
Rat IgG2a-AF647	BioLegend	400526	RTK2758	Isotype control for CXCR2
ACSA-2-APC	Miltenyi	130-117-535	IH3-18A3	Flow cytometry
Zombie Aqua	Thermo Scientitic	L34957	n/a	Flow cytometry
Zombie Aqua	BioLegend	423102	n/a	Flow cytometry
tdTomato	OriGene	AB8181	Polyclonal	IF
Alexa Fluor 594 donkey anti-goat	Thermo Fisher	A-11058	n/a	IF
Phosho-STAT1	Cell Signaling Tech	9167	58D6	Western Blot
Phospho-STAT3	Cell Signaling Tech	9145	D3A7	Western Blot
STAT1	Cell Signaling Tech	9175	42H3	Western Blot
STAT3	Cell Signaling Tech	9132	n/a	Western Blot
Beta Actin	Santa Cruz Biotech	sc-47778	C4	Western Blot
IRDye 800CW goat anti-rabbit	Li-Cor	926-32211	n/a	Western Blot
IRDye 680RD goat anti-mouse	Li-Cor	926-68070	n/a	Western Blot
CCL2 (MCP1)	Novus Biologicals	AF-479-SP	Polyclonal	Neutralizing Ab
IFNAR1	Abcam	ab10739	Polyclonal	Neutralizing Ab
IFNβ	R&D Systems	21400-1	Polyclonal	Neutralizing Ab
Mouse IgG1	R&D Systems	MAB002	11711	Isotype control for IFNβ
Normal Goat IgG	Novus Biologicals	AB-108-C	Polyclonal	Isotype control for CCL2 and IFNAR1

Supplementary Table 4: List of Primers used for genotyping

Strain	Sense (5’-3’)	Antisense (3’-5’)
B6.Cg-Tg(Gfap-cre)77.6Mvs/2J	TTAATCCATATTGGCAGAACGAAAACG	CAGGCTAAGTGCCTTCTCTACA
B6(Cg)-Ifnar1$^{tm1.1Ees}$/J	CCGTTTATTCCATTCTACACAGGTAAGA	CTGTTTCCCCATGGATAACTTCGTA
