# Supplementary material for: Type I interferon response in astrocytes promotes brain metastasis by enhancing monocytic myeloid cell recruitment
Source: Nat Commun. 2023 May 6;14:2632. doi: 10.1038/s41467-023-38252-8 (PMC10163863; doi:10.1038/s41467-023-38252-8)
Supplement: Supplementary file 2 — Reporting Summary [file 41467_2023_38252_MOESM2_ESM.pdf]

nature portfolio

Corresponding author(s): Qing Chen

Last updated by author(s): Apr 6, 2023

Reporting Summary

Nature Portfolio wishes to improve the reproducibility of the work that we publish. This form provides structure for consistency and transparency in reporting. For further information on Nature Portfolio policies, see our Editorial Policies and the Editorial Policy Checklist.

Statistics

For all statistical analyses, confirm that the following items are present in the figure legend, table legend, main text, or Methods section.

n/a	Confirmed	
☐	☒	The exact sample size (*n*) for each experimental group/condition, given as a discrete number and unit of measurement
☐	☒	A statement on whether measurements were taken from distinct samples or whether the same sample was measured repeatedly
☐	☒	The statistical test(s) used AND whether they are one- or two-sided<br>*Only common tests should be described solely by name; describe more complex techniques in the Methods section.*
☐	☒	A description of all covariates tested
☐	☒	A description of any assumptions or corrections, such as tests of normality and adjustment for multiple comparisons
☐	☒	A full description of the statistical parameters including central tendency (e.g. means) or other basic estimates (e.g. regression coefficient) AND variation (e.g. standard deviation) or associated estimates of uncertainty (e.g. confidence intervals)
☐	☒	For null hypothesis testing, the test statistic (e.g. *F*, *t*, *r*) with confidence intervals, effect sizes, degrees of freedom and *P* value noted<br>*Give P values as exact values whenever suitable.*
☒	☐	For Bayesian analysis, information on the choice of priors and Markov chain Monte Carlo settings
☒	☐	For hierarchical and complex designs, identification of the appropriate level for tests and full reporting of outcomes
☒	☐	Estimates of effect sizes (e.g. Cohen's *d*, Pearson's *r*), indicating how they were calculated

Our web collection on statistics for biologists contains articles on many of the points above.

Software and code

Policy information about availability of computer code

Data collection	RNA-seq data was aligned using bowtie293 algorithm against hg38 human genome version and RSEM vl.2.12 software94 was used to estimate read counts and RPKM values using gene information from Ensemble transcriptome version GRCh38.p12. Raw counts were used to estimate significance of differential expression difference between two experimental groups using DESeq295.
Data analysis	Gene set enrichment analysis was done using QIAGEN's Ingenuity® Pathway Analysis software (IPA'", QIAGEGEN, www.qiagen.com/ingenuity) using 'Canonical pathways' options. The web version (v2016) of QIAGEN Ingeuity Pathway® Analysis software was used.<br><br>For bulk RNA seq data, bioinformatic tools were used including CIBERSORT, MCP-counter and xCell.<br><br>Single cell RNAseq analysis of clinical samples was performed in R using the Seurat (v4.l) package. A Seurat object was generated using filtered matrices, gene names, and bar codes downloaded for each dataset. For clustering, we used the first 30 PCs to visualize clusters by uniform manifold approximation and projection (UMAP). We used the first 5 PCs to construct the shared nearest neighbor graph and used a resolution of 0.5 to optimize the modularity function and define clusters. Cell type annotations for each cluster was conducted manually using published gene signatures for myeloid cells.

For manuscripts utilizing custom algorithms or software that are central to the research but not yet described in published literature, software must be made available to editors and reviewers. We strongly encourage code deposition in a community repository (e.g. GitHub). See the Nature Portfolio guidelines for submitting code & software for further information.

Data

Policy information about availability of data

All manuscripts must include a data availability statement. This statement should provide the following information, where applicable:

- Accession codes, unique identifiers, or web links for publicly available datasets
- A description of any restrictions on data availability
- For clinical datasets or third party data, please ensure that the statement adheres to our policy

Data availability statement: The RNAseq data generated in this study have been deposited in the NCBI GEO database under accession code GSE224302. Single cell RNAseq data used for meta-analysis are available from the NCBI GEO database under accession codes GSE174401, GSE176078, and GSE186344. Bulk RNAseq data used for meta-analysis are available from the NCBI GEO database under accession codes GSE125989 and GSE14020. Additional bulk RNAseq data were obtained after requesting access (EGAD00001005046 and PMC6449168) from the corresponding authors of those studies.

Research involving human participants, their data, or biological material

Policy information about studies with human participants or human data. See also policy information about sex, gender (identity/presentation), and sexual orientation and race, ethnicity and racism.

Reporting on sex and gender	N/A
Reporting on race, ethnicity, or other socially relevant groupings	N/A
Population characteristics	N/A
Recruitment	N/A
Ethics oversight	N/A

Note that full information on the approval of the study protocol must also be provided in the manuscript.

Field-specific reporting

Please select the one below that is the best fit for your research. If you are not sure, read the appropriate sections before making your selection.

☒ Life sciences ☐ Behavioural & social sciences ☐ Ecological, evolutionary & environmental sciences

For a reference copy of the document with all sections, see nature.com/documents/nr-reporting-summary-flat.pdf

Life sciences study design

All studies must disclose on these points even when the disclosure is negative.

Sample size	No sample-size calculation was performed. Sample sizes were chosen based on previous experiences with our mouse models and cell lines (e.g. metastasis establishment success rate, expected variance in tumor burden)For in vitro functional assays, at lease 3 samples were included in each group. For in vivo experiments, at least 5 mice included in each group. Individual data point was shown in the graphs.
Data exclusions	No data were excluded
Replication	All the experiments were performed at least 2 times. Numbers of replicates were included in the 'Figure Legends'
Randomization	All samples and experimental mice were randomly distributed into different experimental groups.
Blinding	All the results were collected blindly (BLI, flow, IVM) and quantified by the indicated software unbiasedly.

Reporting for specific materials, systems and methods

We require information from authors about some types of materials, experimental systems and methods used in many studies. Here, indicate whether each material, system or method listed is relevant to your study. If you are not sure if a list item applies to your research, read the appropriate section before selecting a response.

Materials & experimental systems

n/a	Involved in the study	
☐	☒	Antibodies
☐	☒	Eukaryotic cell lines
☒	☐	Palaeontology and archaeology
☐	☒	Animals and other organisms
☒	☐	Clinical data
☒	☐	Dual use research of concern
☒	☐	Plants

Methods

n/a	Involved in the study	
☒	☐	ChIP-seq
☐	☒	Flow cytometry
☒	☐	MRI-based neuroimaging

Antibodies

Antibodies used	List of antibodies are included in 'Supplementary Table 3'. The dilutions for all antibodies have been added to the methods section.
Validation	For flow and immunohistochemical staining, matched Ig controls were used.

Eukaryotic cell lines

Policy information about cell lines and Sex and Gender in Research

Cell line source(s)	Human MDA231-BrM, murine Yumm1.7-BrM, murine A7C11-BrM and murine E0771-BrM cells were generated by in vivo selection. The original Human MDA231, murine A7C11 and murine E0771 cells were generated from female patient or experimental mice. The original murine Yumm1.7 cells were generated from male mice. These information were included in Supplementary Fig. 1c
Authentication	All the BrM cells were authenticated by their abilities to form brain metastatic lesions in vivo. The A7C11 cell line was generated in Jose Conejo-Garcia's lab. We received this cell line directly from his lab. Cell lines from commercial vendors were authenticated by vendors. We did not authenticate A7C11 cells.
Mycoplasma contamination	Cells were routinely tested for mycoplasma with the MycoAlert Detection Kit (Lonza) every 3-6 months at University of Pennsylvania's Cell Center Services. All cell lines used for the experiments tested negative for mycoplasma.
Commonly misidentified lines (See ICLAC register)	No commonly misidentified cell lines were used.

Animals and other research organisms

Policy information about studies involving animals; ARRIVE guidelines recommended for reporting animal research, and Sex and Gender in Research

Laboratory animals	C57BL/6J mice, B6.129-lfnbltmllky/J mice (lfnb-YFP reporter), B6.129S4-Ccr2tmllfc/J (CCR2 knockout), B6.Cg-Tg(Gfapcre)77.6Mvs/2J, and B6(Cg)-lfnarltml.1Ees/J mice were obtained from The Jackson Laboratory. GFAP-Cre line 77.6 mice are particularly useful for selective targeting of astrocytes since they are reported to have no Cre recombinase activity in postnatal or adult neural stem cells (or their progeny) from the hippocampus or other brain regions86. Tg(Gfap-cre)77.6Mvs/2J and B6(Cg)lfnarltml. 1Ees/J mice were crossed to generate Gfap-Cre+/- ; lfnarlf/f and the control Gfap-Cre-/- ; lfnarlf/f mice. The genotyping primers are listed in 'Supplementary Table 4'. Genotyping was performed by Transnetyx, Inc. All experimental animals were used at 5 to 6 weeks of age. Sex of the experimental mice are indicated in the individual experiments. Animals were housed in temperature and humidity controlled environments on a 12-hour light-dark cycle.
Wild animals	No wild animals were used in this study.
Reporting on sex	Both female and male mice were used in the study; results were included; and the sex of the experimental mice were indicated in the figures.
Field-collected samples	No field-collected samples were used in the study.
Ethics oversight	All animal experiments were performed in accordance with protocols approved by the Wistar Institutional Animal Care and Use Committee.

Note that full information on the approval of the study protocol must also be provided in the manuscript.

Flow Cytometry

Plots

Confirm that:

☒ The axis labels state the marker and fluorochrome used (e.g. CD4-FITC).

☒ The axis scales are clearly visible. Include numbers along axes only for bottom left plot of group (a 'group' is an analysis of identical markers).

☒ All plots are contour plots with outliers or pseudocolor plots.

☒ A numerical value for number of cells or percentage (with statistics) is provided.

Methodology

Sample preparation	For single cell preparation for immune cell profiling, brain or lung metastatic lesions and lung tumor nodules were collected and incubated with 200 U/mL collagenase III (Worthington Biochem) for 20 min at 37 °C. The cell pellet was washed by resuspending in fresh MACS buffer (PBS, 0.5% BSA, 2 mM EDTA) and filtering through a 30 ⍰m cell strainer. Spleens were collected, mechanically disaggregated, and filtered over a 70 ⍰m cell strainer. Peripheral blood was collected by live perfusion. Red blood cells in spleen and peripheral blood samples were lysed with ACK lysing buffer.<br><br>For single cell preparation for brain cells, we followed and modified previously published procedures 89,90. In brief, brain metastatic lesions were individually collected and digested with 0.3 U/mL Papain (Worthington Biochem), 40 U/mL DNase I (Worthington Biochem), 0.5 mM EDTA, 1 mM L-cysteine, and 0.067 mM 2-mercaptoethanol for 20 min at 37 °C. Fire polished pipettes were used to physically break down the tissues. After filtering through a 30 µm cell strainer, the cells were resuspended in 0.9 M sucrose and centrifuged at 700 × g for 15 min at 4 °C to remove myelin and debris.<br><br>For astrocyte isolation in brain single cell suspensions, ACSA-2 magnetic microbeads (Miltenyi Biotec) were used following manufacturer's instructions. In brief, cell suspension was incubated with Fc block antibody (CD16/32, 1:10 dilution) for 10 min at 4 °C, followed by incubation with ACSA-2 magnetic microbeads for 15 min at 4 °C. Labeled cells were magnetically sorted on MS columns (Miltenyi Biotec). Both the flow-through (ACSA-2-) and bound fractions (ACSA-2+) were collected for RNA extraction and flow cytometry.<br><br>For identification of immune cells or brain stromal cells, we isolate the brain metastatic lesions based on the BLI signaling. All lesions with total photon flux over 106 were collected for immune cell profiling. Single cell suspensions from brain metastatic lesions were incubated with Fc block (CD16/32, 1:10 dilution) antibody for 15 min on ice. Antibody cocktails (Supplementary Table 3) were diluted 1:100, then added and incubated for 30 min at 4 °C in the dark.
Instrument	Flow cytometry analyses were run on a BD LSRII. Cell sorting was performed on a BD FACSAria II.
Software	All flow data were analyzed in FlowJo (v10.x)
Cell population abundance	Gating strategies to isolate cell types are provided in the figures and supplementary figures. All sorted cells were used immediately in the indicated experiments.
Gating strategy	Preliminary FSC/SSC gates were used to gate against clumped cells as well as to enrich for live cells. Positive staining gating was based on matched Ig controls or unstained samples. Examples of gating were included in the main and supplementary figures.

☒ Tick this box to confirm that a figure exemplifying the gating strategy is provided in the Supplementary Information.
